# Circulating plasma fibronectin affects tissue insulin sensitivity, adipocyte differentiation, and transcriptional landscape of adipose tissue in mice

**DOI:** 10.14814/phy2.16152

**Published:** 2024-07-25

**Authors:** Mahdokht Mahmoodi, Elahe Mirzarazi Dahagi, Mir‐Hamed Nabavi, Ylauna C. M. Penalva, Amrita Gosaine, Monzur Murshed, Sandrine Couldwell, Lisa M. Munter, Mari T. Kaartinen

**Affiliations:** ^1^ Faculty of Dental Medicine and Oral Health Sciences (Biomedical Sciences) McGill University Montreal Quebec Canada; ^2^ Department of Anatomy and Cell Biology, Faculty of Medicine and Health Sciences McGill University Montreal Quebec Canada; ^3^ Department of Pharmacology & Therapeutics, Faculty of Medicine and Health Sciences McGill University Montreal Quebec Canada; ^4^ Centre de Recherche en Biologie Structurale (CRBS) McGill University Montreal Quebec Canada; ^5^ Shriners Hospital for Children Montreal Quebec Canada; ^6^ Department of Medicine (Division of Experimental Medicine), Faculty of Medicine and Health Sciences McGill University Montreal Quebec Canada

**Keywords:** adipose tissue, beige adipocyte, extracellular matrix, insulin sensitivity, obesity, plasma fibronectin

## Abstract

Plasma fibronectin (pFN) is a hepatocyte‐derived circulating extracellular matrix protein that affects cell morphology, adipogenesis, and insulin signaling of adipocytes in vitro. In this study, we show pFN accrual to adipose tissue and its contribution to tissue homeostasis in mice. Hepatocyte‐specific conditional *Fn1* knockout mice (*Fn1*−/−ALB) show a decrease in adipose tissue FN levels and enhanced insulin sensitivity of subcutaneous (inguinal), visceral (epididymal) adipose tissue on a normal diet. Diet‐induced obesity model of the *Fn1*−/−ALB mouse showed normal weight gain and whole‐body fat mass, and normal adipose tissue depot volumes and unaltered circulating leptin and adiponectin levels. However, *Fn1*−/−ALB adipose depots showed significant alterations in adipocyte size and gene expression profiles. The inguinal adipose tissue on a normal diet, which had alterations in fatty acid metabolism and thermogenesis suggesting browning. The presence of increased beige adipocyte markers *Ucp1* and *Prdm16* supported this. In the inguinal fat, the obesogenic diet resulted in downregulation of the browning markers and changes in gene expression reflecting development, morphogenesis, and mesenchymal stem cell maintenance. Epididymal adipose tissue showed alterations in developmental and stem cell gene expression on both diets. The data suggests a role for pFN in adipose tissue insulin sensitivity and cell profiles.

## 1 INTRODUCTION

Adipose tissue (AT) plays an essential role in maintenance of general health via sustaining whole‐body energy metabolism, nutritional homeostasis, and adipokine production (Cristancho & Lazar, 2011). White adipose tissues (WAT) represents energy storage depots (Rosen & Spiegelman, 2014; Zwick et al., 2018), whereas brown adipose tissue (BAT) is thermogenic with a high density of mitochondria allowing for heat generation from fatty acids (Wang & Seale, 2016). Although subcutaneous and visceral WAT both expand in obesity, an increase in the visceral fat around internal organs has more detrimental effects on metabolic health (Wronska & Kmiec, 2012; Zwick et al., 2018). Visceral adipocytes are larger and metabolically more active (more prone to lipogenesis/lipolysis), whereas adipocytes in subcutaneous depots are smaller. Subcutaneous AT is more cellularized, more vascularized, and less prone to inflammation in obesity (Tandon et al., 2018; Wronska & Kmiec, 2012). It also hosts greater amount of mesenchymal‐type stem cells than visceral AT and has capacity to convert to thermogenic beige fat (Si et al., 2019; Wang & Seale, 2016).

AT expansion occurs upon energy surplus via processes involving adipocyte proliferation (hyperplasia), adipogenesis (adipocyte differentiation), lipogenesis within adipocytes, and adipocyte enlargement (hypertrophy) all of which are regulated by insulin (Cristancho & Lazar, 2011; Ghaben & Scherer, 2019). Adipocyte lineage cells are insulin sensitive, however, preadipocytes, smaller adipocytes, and beige adipocytes are more insulin responsive compared to enlarged hypertrophic adipocytes (Acosta et al., 2016; Ghaben & Scherer, 2019; Tandon et al., 2018). Whole‐body and AT insulin sensitivity and signaling are physiologically modulated by a plethora of factors including circulating myokines, adipokines, and cytokines (Ahima & Lazar, 2008; Beale, 2013; Hotamisligil et al., 1996; Zierath, 2002). Locally, in AT, adipocyte insulin sensitivity and signaling can be regulated by the cellular microenvironment and extracellular matrix (ECM) that provides appropriate, lineage stage‐specific adhesion modes and stiffness‐mediated signals to boost insulin signaling (Liu et al., 2005; Poulos et al., 2016). ECM can also affect tissue cell pools to favor the presence of more insulin‐sensitive cells, such as preadipocytes, beige adipocytes, and adipose stem cells. Intracellular insulin signals culminate to AKT (protein kinase B) phosphorylation, which leads to glucose transport and uptake to cells (Beale, 2013; Petersen & Shulman, 2018).

Fibronectin is a multifunctional ECM glycoprotein encoded by *Fn1* gene, which is expressed by multiple cell types as several isoforms (Maurer et al., 2015; Pankov & Yamada, 2002; Schwarzbauer & DeSimone, 2011; Singh et al., 2010). Global deletion of *Fn1* gene in mice results in early embryonic lethality due to defective development of heart and vascular system (George et al., 1997). Two physiological pools of FN exist: circulating plasma FN (pFN) and cellular FN that is made by tissue‐resident cells (Pankov & Yamada, 2002; To & Midwood, 2011). pFN is synthesized by hepatocytes in the liver and secreted into the bloodstream, where it circulates at a high concentration of 200–600 μg/mL in humans and 100–400 μg/mL in mice (Maurer et al., 2015). pFN does not contain any extra domains that are frequently detected in cellular FN (cFN) (Tamkun & Hynes, 1983; To & Midwood, 2011). Previous research indicates that circulating pFN can absorb into tissues to form ECM fibrils, and which contributes to tissue integrity (Moretti et al., 2007; Oh et al., 1981). Experiments where pFN has been labeled and injected into rodent models or genetically deleted have demonstrated that pFN integrates to and/or has roles in skin, kidney, liver, heart muscle, bone, lung, vasculature and brain (Kumra et al., 2018; McKeown‐Longo & Mosher, 1983; Moretti et al., 2007; Oh et al., 1981), where it may form up to 90% of the total FN matrix (Bentmann et al., 2010). It has not been reported if pFN contributes to AT or other metabolic tissue ECM in vivo, however, the important role of FN in general in adipocyte biology has been demonstrated via in vitro studies showing that FN ECM sustains preadipocyte phenotype and inhibits adipose conversion jointly with preadipocyte factor‐1 (Pref1) (Chernousov et al., 1991; Fukai et al., 1993; Hudak & Sul, 2013; Kamiya et al., 2002; Sul et al., 2000; Wang et al., 2010). Our work has also shown that pFN, from cell culture serum, readily forms ECM and promotes preadipocyte proliferation and potentiates the pro‐proliferative effects of insulin (Myneni et al., 2014). The decrease of pFN ECM in turn promotes proadipogenic insulin signaling (Myneni et al., 2014). Furthermore, we showed that pFN assembly in adipocyte cultures is promoted by transglutaminase (TG) activity from plasma TG, Factor XIII‐A (FXIII‐A), which is produced by preadipocytes (Myneni et al., 2014). FXIII‐A null AT has increased insulin sensitivity and decreased FN and collagenous ECM (Myneni et al., 2016). The role of pFN in vivo in whole‐body metabolism or in AT function has not been explored. In this study, we have investigated the potential contribution of circulating pFN to WAT and metabolism in vivo. We report that pFN adsorbs from blood to subcutaneous (inguinal WAT), and visceral (epididymal WAT), and BAT as well as other metabolic tissues such as liver and skeletal muscle. The elimination of FN in hepatocytes in a mouse model (a hepatocyte‐specific *Fn1* conditional knockout (*Fn1*−/−ALB)) enhances glucose clearance, whole‐body and tissue insulin sensitivity in mice on a normal diet. The phenotype is particularly evident in the subcutaneous WAT where markers of beige adipocytes were detected. Induction of weight gain via high‐fat diet (HFD) feeding revealed no change in weight or fat depot volumes in *Fn1*−/−ALB mice but caused a change in transcriptional profiles reflecting developmental genes and maintenance of stem cells. Our work assigns a novel function to pFN in tissue insulin sensitivity and AT cell homeostasis.

## 2 METHODS

### 2.1 Reagents and antibodies

Antibodies against pAKT (Ser473) (Rabbit monoclonal, Cat# 4060S), pAKT (Thr308) (Rabbit monoclonal, #13038S) and AKT (pan) (Rabbit monoclonal, Cat# 4691S) and antibody against β‐actin (Rabbit polyclonal, Cat# 4967S) were from Cell Signaling Technology (Danvers, MA, USA). Antibodies against fibronectin; mouse monoclonal (Cat# ab632) and rabbit polyclonal (Cat# ab2413) were from Abcam (Cambridge, UK). FXIII‐A antibody (SAF13A‐AP, sheep polyclonal) was from Affinity Biologicals (Ancaster, ON, Canada). Dulbecco's Modified Eagle Medium (DMEM), GlutaMAX (12561‐056), penicillin–streptomycin, and sodium pyruvate were purchased from Gibco (Burlington, ON, Canada). Fetal bovine serum was obtained from Hyclone (Waltham, MA, USA). All other reagents if not defined were purchased from Sigma‐Aldrich.

### 2.2 Animals

C57/bl6 male mice (for plasma FN injections) were purchased from The Jackson Laboratory (Bar Harbor, ME, USA). Liver‐specific deletion of *Fn1* gene results in elimination of FN from circulation. Plasma FN knockout model (C57/bl6 background) was generated as before via breeding *Fn1*flx/flx and ALB‐Cre driver mouse. The *Fn1* flx/flx mice, originally developed by Sakai et al. (2001) were generously provided to us by Dr. Faessler from Max Plank Institute for Biochemistry (Munich, Germany) via Dr. Reinhardt from McGill University (Montreal, Canada) (model also available at Jackson Laboratory; JAX#029624). ALB‐Cre mouse (JAX# 003574) (Postic et al., 1999) was purchased from The Jackson Laboratory (Bar Harbor, ME, USA). All animals were housed in a pathogen‐free animal facility and kept on a diurnal cycle and had ad libitum access to food and water. The animal care and experimental procedures were under the Canadian Council of Animal Care and approved by the McGill University Animal Care Committee.

### 2.3 Plasma fibronectin labeling and detection in vitro and in vivo

pFN (human plasma FN (Cat# FC010; Millipore, Burlington, MA, USA)) was labeled with AlexaFluor568 or Alex aFluro680 according to instructions of the manufacturer (ThermoFisher, Waltham, MA, USA). Briefly, pFN was concentrated to 1 mg/mL solution with Amicon® Ultra 3 K Centrifugal Filter Devices (3000 molecular weight cut off) (Sigma‐Aldrich, St Louis, MI, USA), followed by two sequential dialysis processes using dialysis cassettes (Slide‐A‐Lyzer™ Dialysis Cassettes, 10 K MWCO) (ThermoFisher, Waltham, MA, USA) against 100 mM NaHCO_3_ + 500 mM NaCl pH 8.4 buffer. Protein concentration was measured using Micro BCA Protein Assay Kit (ThermoFisher, Waltham, MA USA). Dissolved AlexaFluor 568 NHS Ester (Invitrogen, Waltham, MA USA) in dimethyl sulfoxide (DMSO) at concentration of 10 mg/mL was added to the pFN solution, and the solution was incubated at room temperature (RT) for 1 h with constant and gentle shaking. The unbound dye was removed via two dialysis steps (as above) at 4°C against 100 mM NaHCO_3_ + 500 mM NaCl, pH 8.4. BCA assay was used to determine the final protein concentration of AlexaFluor 568‐pFN. Labeling degree was assessed according to the manufacturer's recommendations and determined to be 3.5 AF568 molecules per one pFN molecule. The ability of pFN to create a fibrillar matrix, was confirmed in 3 T3‐L1 cells via adding AlexaFluor568‐pFN or AlexFluro680 to media at 2 μg/mL concentration to (see below).

### 2.4 3T3‐L1 cultures

3T3‐L1 Mouse embryonic fibroblasts (MEFs) (ATCC, State, USA) cells were cultured in 8‐well Nunc Lab‐Tek® II glass chamber slides (Fisher Scientific) in standard cell culture conditions (CO_2_ 5%, temperature 37°C with saturating humidity). Dulbecco's Modified Eagle Medium (DMEM) with 10% of FBS (fetal bovine serum) and 1% of penicillin, and streptomycin was used for cell culture medium. AlexaFluor568‐pFN or AlexaFluor680‐pFN (2 μg/mL) was added to the media on day 7 for 24 h after which cells were washed and fixed with 3.7% formalin for 10 min at RT followed by permeabilization with 0.25% Triton X‐100 for 10 min. Cells were then blocked with 2% BSA. DAPI was used to stain the nuclei. Fluorescence images were captured with AxioImager M2 microscope and Orca Flash 4.0 camera (Zeiss).

### 2.5 pFN labeling and injections and detection in tissues

pFN (Cat# FC010; Sigma) was labeled with AlexaFluor 680 (AF680) as described above. Two groups of 12‐week‐old C57/bl6 male mice (*n* = 3 for each group) (The Jackson Laboratory, Bar Harbor, ME, USA) were used. The experimental group received 20 mL/kg of AF680‐pFN solution (i.e., 1 mg pFN) via intraperitoneal (IP) injections while the control group was injected with inactivated AF680 that corresponded to the degree of pFN labeling (3.5 AF680 per pFN). Inactivation was done by mixing the AF680 with 0.5 M NaCl in 50 mM Tris, pH 8. This injection regimen was repeated on three consecutive days, with alternating injection sites. After 24 h following the final injection, the mice were euthanized using CO_2_ asphyxiation under isoflurane anesthesia, and following tissues were collected: plasma, liver, white visceral adipose tissue (epididymal or periovarian, eWAT); white subcutaneous adipose tissue (inquinal, iWAT); brown fat, pancreas, bone marrow, and skeletal muscle (quadriceps femoris). iWATand eWAT were imaged immediately ex vivo. All tissues were stored at −80°C for further analyses. Ex vivo epifluorescence imaging of tissues was done with PerkinElmer IVIS Spectrum In Vivo Imaging System (PerkinElmer, Waltham, MA, USA) at Mouse Housing Facility of The Rosalind and Morris Goodman Cancer Institute at McGill University. For fluorometric quantification, tissues were extracted with sodium dodecyl sulfate (SDS) and deoxycholic acid (DOC) and TritonX‐100 containing buffer for maximal FN solubilization (Makino et al., 1973; McKeown‐Longo & Mosher, 1984) (150 mM NaCl, 10 mM Tris–HCl, 5 mM EDTA, 0.1% SDS, 1.0% Triton X‐100, 1.0% Na‐Deoxycholate pH 7.2). The fluorescence intensity in extracts was measured with Tecan Fluorometric Plate Reader Infinite 200 (Tecan, Männedorf, Switzerland) with Ex/Em 560 nm/610 nm and expressed as mg of AF680‐pFN per mg of total extracted protein (measured by BCA assay). The quantity of AF680‐pFN was determined via the AF680 standard curve considering a 3.5/1 labeling degree. Plasma AF680‐pFN gels were imaged with Molecular Dynamics/Amersham Typhoon 8600 Variable Mode Imager (Molecular Dynamics/Amersham Biosciences, Amersham, UK) or Odyssey M Imaging System (Li‐Cor, Lincoln, NE, USA).

### 2.6 Diet‐induced obesity model

Diet‐induced obesity model was generated from male and female *Fn1*−/−ALB mice and their littermate, sex‐matched control *Fn1*flx/flx mice (*n* = 5–11) with a 20‐week special feeding regime of ad libitum high‐fat diet (HFD)‐ (Ranea‐Robles et al., 2022) (60% fat/lard and soybean oil) (Cat# TD.06414) or nutrition‐matched control diet (CD) (10% fat) (Cat# TD.08806) (Inotiv Inc., West Lafayette, Indianapolis, IN, USA). The weight of the mice was assessed weekly. At the endpoint, mice were assessed for insulin and glucose tolerance and fat mass (as below). Adipose tissues: inguinal, subcutaneous adipose tissue (iWAT) and epidydimal or periovarian, visceral adipose tissue (eWAT), liver, and skeletal muscle (quadriceps femoris) were collected for further analysis.

### 2.7 Computed tomography (CT) scans and dual‐energy X‐ray absorptiometry (DEXA)‐analysis for in vivo fat mass

All CT scan experiments were performed on nanoScan SPECT/CT for small animals (Mediso Medical Imaging Systems, Budapest, Hungary) at Small Animal The Small Animal Imaging Labs (*SAIL*) at Research Institute of McGill University Health Centre. The CT scans were conducted under anesthesia (Isoflurane 2%, medical air [0.6 L/min]) delivered by a nose cone. Temperature and heart rate were monitored throughout the procedure using a Mediso system. The protocol was performed using 480 number of projections, scan method helical, 50 kVp, 610 μA, expose time 8 min and voxel size 250 μm. The reconstructed CT image was segmented automatically into the visceral adipose tissue (eWAT) and the subcutaneous adipose tissue (iWAT) using a MATLAB based software developed in‐house. MATLAB's Canny algorithm was used to enhance the edge of the wall in order to separate the eWAT and iWAT into two independent volumes. In the case this failed for some animals when the image of the abdominal muscular wall was not completely recognizable by MATLAB, we used the software *fsleyes* to conduct the segmentation along the muscular wall manually. Total tissue mass (g), lean mass (g) and fat mass (%) were analyzed with dual‐energy X‐ray absorptiometry (DEXA) analysis at the Centre for Bone and Periodontal Research at McGill University. The DEXA scans were conducted under ketamine anesthesia using Lunar PIXImus II Mouse Densitometer (GE Medical Systems, Madison, WI) with 0.3 mm stationary anode X‐ray tube and following parameters: Filtration Nom: 2.5 mm Al (@70 kV), Focal spot size: 0.25 mm × 0.25 mm; Imaging area 100 mm × 80 mm; Max. X‐ray tube voltage: 80 kV; Max. X‐ray tube current: 500 μA. After scanning, the fat % was analyzed by using custom‐defined ROI function.

### 2.8 Glucose tolerance test (GTT) and insulin tolerance test (ITT)

GTT and ITT were done at the endpoint of the experiment at 20 weeks on CD or HFD groups after 6 h of fasting. For the GTT, mice were given IP injections of glucose (1 g/kg body weight), and blood glucose level was measured using a glucometer (FreeStyled Life, Abbot) at 0, 15, 30, 60, and 120 min after glucose injection. For the ITT, insulin (0.8 U/kg body weight) (Humulin® N, Lilly, Canada) was injected, and blood glucose levels were measured at 0, 30, 60, 90, and 120 min after insulin injection. The area under the curve was calculated using GraphPad Prism versus 10. kITT (% decrease of glucose per minute) (Okita et al., 2014) was calculated from 0 to 30 min data points (Monzillo & Hamdy, 2003) presented as graph created with GraphPad Prism vs 10.

### 2.9 In vivo and in vitro insulin sensitivity of adipose tissue

Insulin sensitivity of iWAT and eWAT was conducted via in vivo insulin injections and quantification of phosphorylation ratio in signaling pathways via Western blotting. *Fn1*flx/flx and *Fn1*−/−ALB mice on a CD and HFD were fasted for 6 h and given IP injections of insulin (1–2 U/kg) (Humulin® N, Lilly, Canada). Mice were euthanized 15 min postinjection followed by dissecting iWAT (inguinal fat) (subcutaneous) and eWAT (perigonadal fat; epididymal) (male) and periovarian (female) representing visceral adipose tissue and homogenized in extraction buffer. The extraction buffer contains 100 mM Tris (pH 7.4), 1% Triton X‐100, 10 mM EDTA, 100 mM sodium fluoride, 2 mM phenylmethyl sulfonyl fluoride, 5 mM sodium orthovanadate, in addition to phosphatase inhibitor and protease inhibitor cocktail. The protein lysate was analyzed by Western blotting as described below. The blots were probed with anti‐Phospho‐AKT (Ser473 and Thr308)/anti‐total‐AKT. Quantification of band intensities was done with the NIH Image J open‐source image‐processing program.

### 2.10 Quantitative RT‐PCR (qPCR) for adipocyte markers

mRNA was extracted using the RNeasy Mini Kit (Cat# 74106, Qiagen, Venlo, the Netherlands) and cDNA prepared using the High‐Capacity cDNA Reverse Transcription Kit (Cat# 4368814, Applied Biosystems, Foster City, CA). qPCR was done on the StepOnePlus Real‐Time PCR System (Applied Biosystems) in a 20 μL reaction volume consisting of the following: 9 μL (50 ng) synthesized cDNA, 10 μL TaqMan Fast Advanced Master Mix and 1 μL of each TaqMan Gene Expression Assay. TaqMan® Fast Advanced Master Mix (Cat# 4444557) and primers were purchased from Applied Biosystems. Following primers were used: *Pref1* (Mm00494477_m1), *Ucp1* (Mm01244861_m1), *Prdm16* (Mm00712556_m1). Expression was normalized to Gapdh (Mm99999915_g1).

### 2.11 Leptin and adiponectin

Plasma was collected at end point of each feeding regime after 6 h fasting. Leptin and adiponectin levels were measured with Mouse Leptin ELISA Kit (Cat# ab100718) and Mouse Adiponectin ELISA Kit (Cat# ab108785) (Abcam, Cambridge, UK) according to the instruction of the manufacturer.

### 2.12 Protein extraction, SDS‐PAGE and Western blotting

Protein extractions were prepared using sodium dodecyl sulfate (SDS) and deoxycholic acid (DOC) and TritonX‐100 containing buffer for maximal FN solubilization (Makino et al., 1973; McKeown‐Longo & Mosher, 1984) (150 mM NaCl, 10 mM Tris–HCl, 5 mM EDTA, 0.1% SDS, 1.0% Triton X‐100, 1.0% Na‐Deoxycholate pH 7.2) containing a protease inhibitor and phosphatase inhibitor cocktails (Sigma). Protein concentrations were determined using the BCA protein assay kit (Pierce, Rockford, IL, USA). For Western blotting, proteins were resolved by 8.5% SDS‐PAGE gel electrophoresis and transferred to PVDF membranes (Bio‐Rad, Mississauga, ON, Canada). Membranes were blocked with 5% nonfat milk powder in Tris‐buffered saline‐Tween (TBS‐T) buffer, and each protein was detected with specific antibodies followed by corresponding HRP‐conjugated secondary antibodies. The detection was visualized with Amersham ECL Prime Western Blotting Detection Reagent (GE Healthcare Life Sciences, Baie d'Urfe, QC, Canada). Quantification of band intensities (where indicated) was done with the NIH Image J open‐source image‐processing program. Albumin was visualized in SDS‐PAGE gels via Coomassie staining.

### 2.13 Adipose tissue histology

At the endpoint of HFD and CD‐feeding *Fn1*flx/flx and *Fn1*−/−ALB mice iWAT and eWAT were collected for histological analysis. Tissues were fixed in 3.7% formalin, paraffin‐embedded, sectioned, and stained with hematoxylin & eosin (H&E) staining. The number and size of cells were quantified using open‐source image analysis software, CellProfiler (https://cellprofiler.org/) (McQuin et al., 2018; Stirling et al., 2021) via a systematic assessment of cell size distribution from 5 separate fields of microscopic image per genotype. The workflow using CellProfiler or ImageJ, with a focus on the “*Adipocytes_Sizing_Pipeline*.” In the CellProfiler pipeline, the *ColorToGray* module initially converted the color images to grayscale, followed by the transformation into a binary black‐and‐white representation using the *ImageMath* module. Subsequent modules, including *EnhancedOrSuppress* Features, *EnhanceEdges*, and Morph, were strategically applied to enhance membrane visibility and facilitate adipocyte distinction by eliminating specks and sharpening edges. The *IdentifyPrimaryObjects* module was used to delineate adipocyte membranes based on the enhancements achieved in prior steps. The subsequent *CovertObjectsToImage* module integrated these identified objects back into the original black‐and‐white image, resulting in a clearer representation of the cell membranes. To specifically focus on adipocytes, another round of processing was conducted using *ImageMath* and *EnhancedOrSuppress* Features modules. The *IdentifyPrimaryObjects* module was then reapplied, this time recognizing adipocytes as objects. In *Test Mode*, the module allowed visual confirmation of adipocyte identification, providing flexibility for manual adjustments to meet specific preferences.The *MeasureObjectSizeShape* module quantified key parameters such as the area of each adipocyte, generating raw measurements in pixels. To convert these measurements into microns, the *CalculateMath* module was utilized, incorporating a scaling diagram approach. This involved determining the pixel‐to‐micron conversion factor using the scaling diagram present in each image. Aa 10× magnification, the conversion factor was calculated as 0.13516 μm^2^/pixel^2^, enabling accurate transformation of pixel‐based area measurements into meaningful micrometer values. Adipose cell size was reported based on the surface area of detected cells up to 10,000 μm^2^.

### 2.14 RNA sequencing and data analyses

The total RNA was extracted from iWAT and eWAT of CD‐ and HFD‐fed mice and their control groups. RNA sequencing process was done at Genome Quebec Next Generation Sequencing Platform as follows: Total RNA was quantified, and its integrity was assessed on a LabChip GXII (PerkinElmer) instrument. Libraries were generated from 250 ng of total RNA as following: mRNA enrichment was performed using the NEBNext Poly(A) Magnetic Isolation Module (New England BioLabs). cDNA synthesis was achieved with the NEBNext RNA First Strand Synthesis and NEBNext Ultra Directional RNA Second Strand Synthesis Modules (New England BioLabs). The remaining steps of library preparation were done using and the NEBNext Ultra II DNA Library Prep Kit for Illumina (New England BioLabs). Adapters and PCR primers were purchased from New England BioLabs. Libraries were quantified using the KAPA Library Quantification Kits—Complete kit (Universal) (Kapa Biosystems). Average size fragment was determined using a LabChip GXII (PerkinElmer) instrument. The libraries were normalized and pooled and then denatured in 0.05 N NaOH and neutralized using HT1 buffer. The pool was loaded at 175 pM on a Illumina NovaSeq S4 lane using Xp protocol as per the manufacturer's recommendations. The run was performed for 2 × 100 cycles (paired‐end mode). A phiX library was used as a control and mixed with libraries at 1% level. Base calling was performed with RTA v3.4.4. Program bcl2fastq2 v2.20 was then used to demultiplex samples and generate fast reads. The data was analyzed with R Statistical Software (https://www.r‐project.org/) (R Core Team, 2020) in R Studio and results were plotted using ggplot2, ggrepel and dplyr libraries from comprehensive R archive network (CRAN). Differentially expressed genes (DEGs) in iWAT and eWAT, between pFN KO and flx/flx mice under the two different diets were analyzed. Transcripts were considered differentially expressed when the absolute value of the log‐fold change had the *p*‐value than 0.05. Gene enrichment analysis was done with Metascape (https://metascape.org) and selected overrepresented GOterms were confirmed via analysis using Panter database (https://www.pantherdb.org/) and KEGG (Kyoto Encyclopedia of Genes and Genomes) (https://www.genome.jp/kegg/).

### 2.15 Statistical and data analysis

Data were analyzed with GraphPad Prism software (version 10). Results were expressed as mean ± SD (standard deviation). Data were analyzed by the one‐way and two‐way analysis of variance (ANOVA) followed by multiple comparisons via Tukey post‐hoc test. When comparing two groups Student's *t*‐test was used. The differences are considered statistically significant for *p*‐values < 0.05. *p*‐values are noted in each Figure.

## 3 RESULTS

**FIGURE 1:**
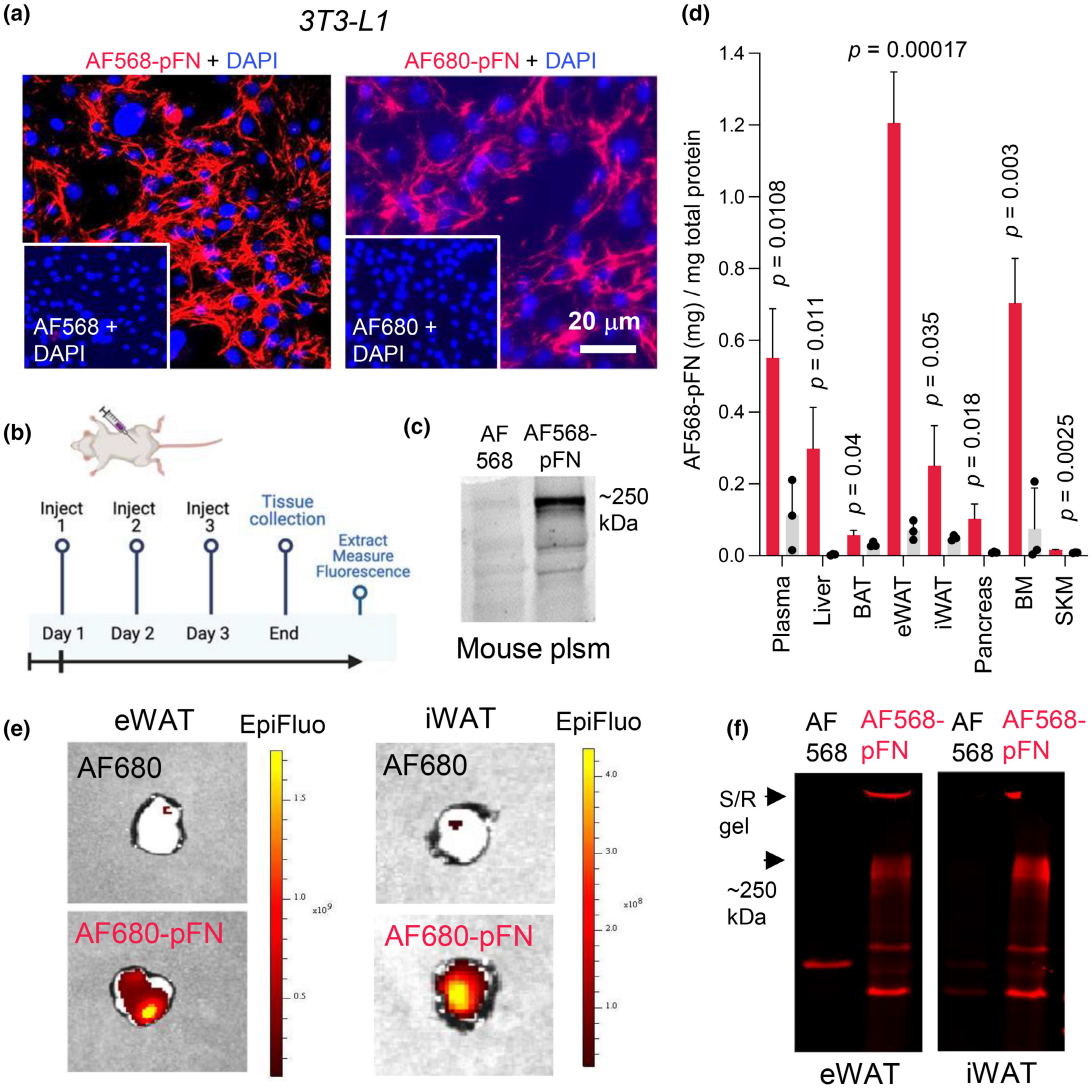
pFN accumulates from circulation to metabolic tissues. (a) AlexaFluor568 (AF568)‐pFN and AlexaFluor680 (AF680)‐pFN were confirmed to undergo fibrillogenesis in 3T3‐L1 MEF cell cultures. Inactivated AF‐fluorophores were used as controls and did not show integration to cell layers. (b) Injection regime involved three consecutive intraperitoneal injections of 1 mg of labeled pFN per day. Control mice received inactivated label corresponding to the labeling degree (3.5 AF‐fluorophores in each pFN). Image was created with BioRender.com. (c) Mouse plasma (20 µg total protein) analyzed by SDS‐PAGE and visualized by fluoroimager shows the presence of AF568‐pFN circulation after injections. (d) Levels of AF568‐pFN in plasma, liver, BAT (brown adipose tissue), eWAT, iWAT, pancreas, bone marrow (BM) and skeletal muscle (SKM) were quantified by fluorometric analyses after SDS‐DOC‐TritonX100 extractions of the tissues. Graph demonstrates accrual of pFN to metabolic tissues, highest levels detected in eWAT. Plasma (plsm) is visualized as control. Error bars represent SD. *n* = 3 (WT male mice). Statistical significance is expressed as *p*‐values comparing AF568‐pFN versus AF568. (e) Ex vivo imaging AF680‐pFN accumulation to eWAT and iWAT. Imaging was done with IVIS Spektrum immediately after dissecting the tissues. (f) Accumulation of AF568‐pFN was further visualized via SDS‐PAGE of iWAT and eWAT extracts. Full length AF568‐pFN is detected at 250 kDa with some high molecular weight material at the border of stacking and running gel (S/R gel).

pFN circulates in the blood at high concentration and contributes to ECM of several tissues. In this study, we have examined its potential contribution to AT and metabolism. We first examined if circulating pFN accumulates from blood to metabolic tissues; eWAT, iWAT, BAT, liver, pancreas, bone marrow and skeletal muscle via labeling pFN with AlexFluor568 (AF568‐pFN) and AlexaFluro680‐pFN (AF680‐pFN) followed by intraperitoneal (IP) injections to normal, adult mice. Prior to IP injections, we confirmed in 3T3‐L1 preadipocyte cell cultures that the fluorescence labeling does not interfere with the ability of pFN to create a fibrillar matrix and that the inactivated AF‐label alone does not integrate into cell layers (Figure 1a). The in vivo study involved an injection regime of three IP injections in consecutive days (like we and others have done before; Figure 1b) (Bentmann et al., 2010; Kumra et al., 2018; Mousa et al., 2017). Control injection group included inactivated AF568 or AF680 label that corresponded to the labeling degree that was calculated for AF‐pFN. After the three injections, the presence of intact full length AF568‐pFN in plasma was detected by SDS‐PAGE gels and fluoroimaging (Figure 1c). At the end of the experiment, plasma was collected and metabolic tissues (plasma, liver, eWAT [epididymal fat = visceral]; iWAT [inguinal fat = subcutaneous]; BAT [brown fat], pancreas, bone marrow, and skeletal muscle [femoral quadriceps]) were dissected, and proteins extracted using SDS‐DOC‐Triton X‐100 containing buffer to solubilize ECM. AF568 fluorescence was measured with a fluorometer and expressed as μg of pure labeled pFN‐568 per mg of tissue (determined from a standard curve) (Figure 1d). Significant 568 fluorescence (compared to control inactivated AF568 injected groups) was detected in plasma, liver, iWAT, eWAT and bone marrow was observed. BAT, pancreas and skeletal muscle also showed AF568‐pFN accumulation but markedly less than others assessed (Figure 1d). AF568‐pFN accumulation in eWAT and iWAT were also confirmed with ex vivo epifluorescence imaging (Figure 1e) and via SDS‐gels of the extracts followed by fluoroimaging (Figure 1f). These data demonstrate that pFN is a component of AT depots and suggests a role in AT function.

**FIGURE 2:**
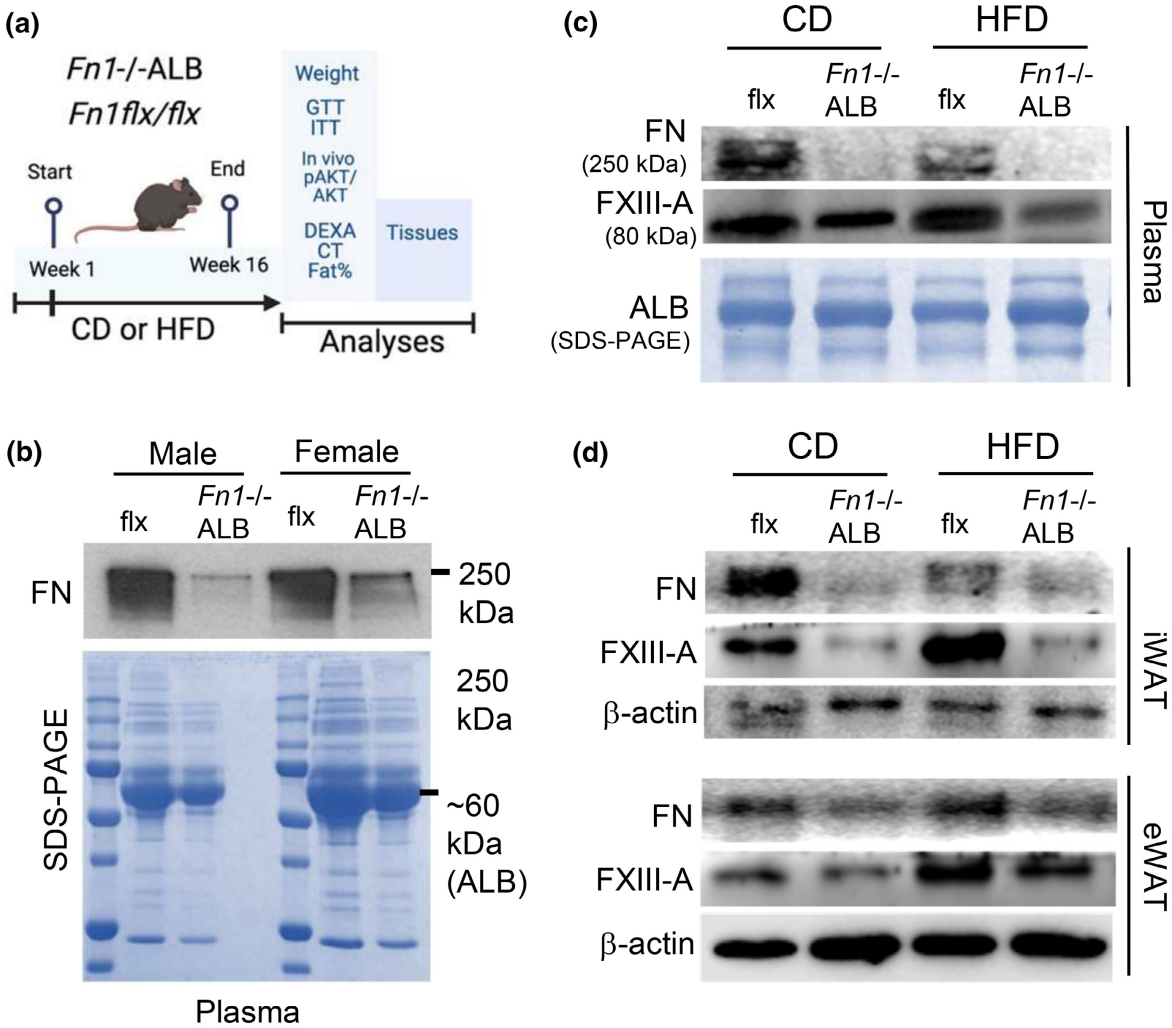
Hepatocyte‐specific fibronectin knockout mice (*Fn1*−/−ALB) knockout mice, aka, plasma FN knockout mice show low FN levels in white adipose tissues (WAT). (a) *Fn1*−/−ALB mice were developed by breeding of *Fn1*flx/flx and ALB‐Cre mice. Mice were fed an obesogenic high‐fat diet (HFD) or nutrition matched control diet (CD) for 20 weeks which was followed by metabolic and adipose tissue phenotyping. Image was created with BioRender.com. (b) Western blot analysis of plasma (1 µg protein/lane) shows efficient elimination of circulating FN in male *Fn1*−/−ALB mice. Females had residual circulating FN. Lower panel shows Coomassie Blue stained SDS‐PAGE of whole plasma protein panel (0.5 µg total protein loaded) where albumin (ALB) shows a strong band. (c) Western blot analysis of FN (250 kDa) and FXIII‐A (80 kDa) levels in plasma of CD‐ and HFD‐fed mice show elimination of FN but no effect on FXIII‐A levels between *Fn1*−/−ALB and its flx control. (d) Western blot analysis of FN and FXIII‐A levels in iWAT (inguinal WAT; subcutaneous AT) and eWAT (epididymal WAT; visceral AT) of CD and HFD‐fed mice show low FN and FXIII‐A content in iWAT. eWAT shows less alterations.

To investigate of pFN affects adipocytes or AT expansion, that is, weight gain or whole‐body metabolism, we developed diet‐induced obesity mouse model in the absence of plasma FN. For this, pFN deficient mice were created via breeding *Fn1*flx/flx and ALB‐Cre driver mice as before (Sakai et al., 2001) (Figure 2a). *Fn1*−/−ALB mice gradually eliminate circulating FN in 2–3 months post‐birth reaching 96% decrease that last at least until 8 months of age (Sakai et al., 2001; Yi et al., 2003). Our model (at 3 months) showed clear decrease in FN in plasma particularly in male mice, and otherwise similar protein pattern in SDS‐PAGE analysis (Figure 2b). Analysis of the fibronectin band intensity gave an estimation of the decrease, which was −93% in male plasma and −79% in female plasma (normalized to albumin in 1 μg of total loaded protein). *Fn1*−/−ALB mice and their littermate controls (flx/flx) of both sexes were placed on obesogenic high‐fat diet (HFD) (60% fat) and nutrition‐matched control diet (CD) (10% fat) for 20 weeks with weekly weight monitoring. Control and knockout, male and female mice had similar weights at 4‐week age prior to beginning the feeding regime (Figure S1). At the end point, when mice were 6 months old, FN levels in plasma and eWAT and iWAT were analyzed for FN (general antibody that detects all FN, including cellular FN) showing clear absence of circulating FN in *Fn1*−/−ALB mice on both diets and no major change in the plasma FN levels between the diets (estimate decrease in the knockout versus control on CD was −94% and on HFD 95%) (Figure 2c). The *Fn1*−/−ALB had similar circulating FXIII‐A and albumin levels (Figure 2c). Interestingly, the iWAT of the *Fn1*−/−ALB mice showed a dramatic decrease of FN content on both diets (estimated decrease in knockout compared to control on CD; −86% and HFD; −45%) suggesting that large portion of AT FN derived from circulation (Figure 2d). Furthermore, FXIII‐A transglutaminase levels showed also a clear decrease in iWAT of the knockout suggesting a regulatory a link between two (Figure 2d). Total FN and FXIII‐A levels in eWAT were not as clearly altered in the knockout compared to iWAT (estimated decrease in knockout on CD; −62% and HFD; −61%).

**FIGURE 3:**
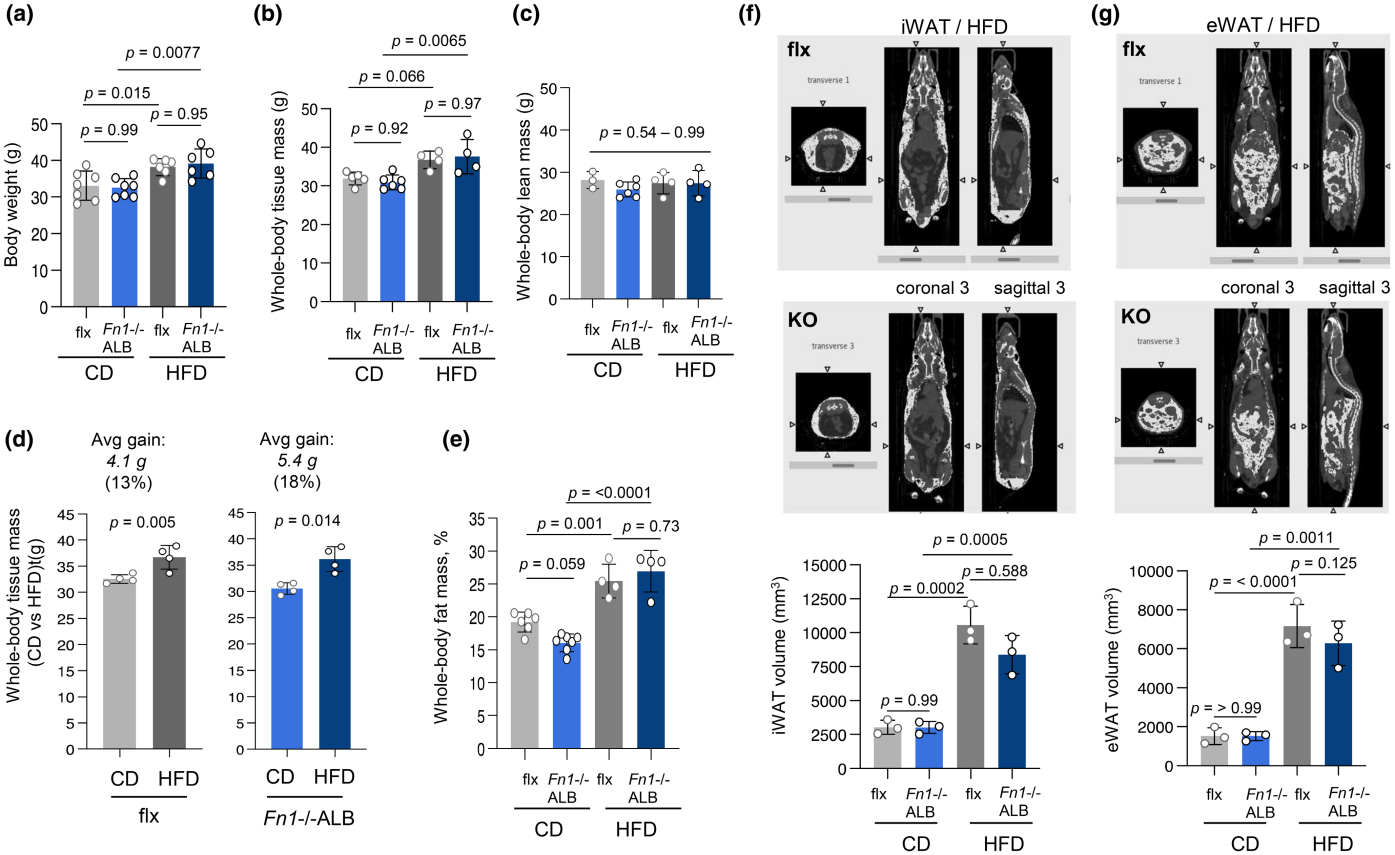
*Fn1*−/−ALB mice gain weight on high‐fat diet (HFD) normally and have normal levels of lean and fat mass. (a) Body weights of male *Fn1*−/−ALB and its control flx mice after 20 weeks on control diet (CD) and high‐fat diet (HFD). Female mice did not show significant weight gain after HFD feeding in control mice (Figure S1). (b, c) Dual‐energy X‐ray absorptiometry (DEXA)‐scans show no significant difference between flx and *Fn1*−/−ALB mice in whole‐body tissue mass nor in lean mass (g). (*n* = 3–4). (d, e) Whole‐body tissue mass from DEXA scans assessing weigh gain shows modest 13% (flx) and 18% (*Fn1*−/−ALB) total mass gain on HFD. Assessment of increase in fat % (fat/total tissue mass) shows significant increase for both flx and *Fn1*−/−ALB mice. (f, g) Computed tomography (CT) scans for iWAT and eWAT volumes show significant increase on HFD, but no significant difference between male flx and *Fn1*−/−ALB mice. CT scan images (transverse, coronal and sagittal images) show equal and similar fat distribution and quantification shows no significant differences. Error bars represent SD (*n* = 3–5 per group). Statistical significance is represented as *p*‐values between knockout and control or CD versus HFD.

Weight and fat mass analysis at the end point of the 20‐week HFD feeding demonstrated that male *Fn1*−/−ALB mice gained weight in a similar manner as the control, flx/flx mice (Figure 3a). While female *Fn1*−/−ALB mice gained significant increase in body weight on HFD, flx/flx female mice did not (Figure S2) and thus further steps of the study were conducted mostly with male mice. Body composition analysis via DEXA scans showed similar total whole‐body tissue mass increase (g) in both flx and *Fn1*−/−ALB mice (Figure 3b) and no change in lean mass (Figure 3c). Weight gain over the course of the 20‐week feeding was modest but significant (average of 4.1 g [13%] increase for flx/flx and 5.4 g [18%] for *Fn1*−/−ALB mice; Figure 3d). DEXA analysis of fat mass (%) (fat/total tissue mass) showed that *Fn1*−/−ALB on CD had notably lower total fat mass % albeit this was not significant (*p* = 0.059) (Figure 3e). Whole‐body CT (computed tomography) scans iWAT and eWAT depots showed a significant 4‐fold increase of both depots, and there was no significant difference between the knockout and control (Figure 3f,g). Reflecting the weigh, fat mass, and adiposity, circulating leptin was significantly increased in both models on HFD; but no alterations were seen in adiponectin levels (Figure S3).

**FIGURE 4:**
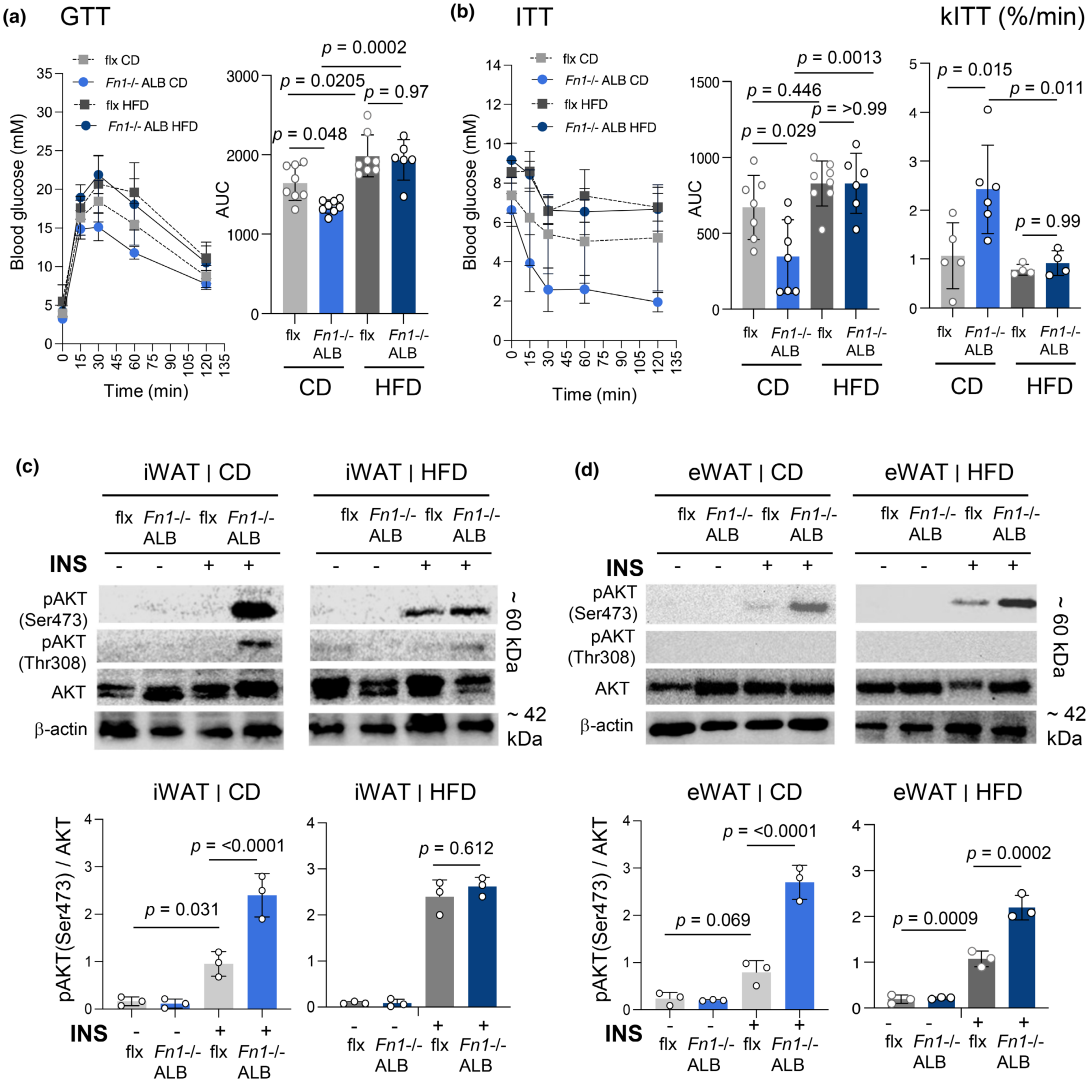
Absence of pFN significantly improves whole‐body glucose homeostasis and significantly increases in vivo insulin sensitivity of adipose tissues of male mice. (a) Glucose tolerance test (GTT) at the end of 20‐week feeding regimes. Significantly enhanced glucose clearance was detected in *Fn1*−/−ALB mice on CD which presented significantly smaller Area Under the Curve (AUC) values. The difference disappeared in HDF. (b) Insulin tolerance test (ITT) showed same trend, knockout mice had significantly smaller AUC, that is, enhanced insulin sensitivity and significantly higher rate of glucose decrease in 30 min (kITT) (glucose decrease% per min). Error bars represent SD (*n* = 6–8 per group). Statistical significance is represented as *p*‐values. (c, d) In vivo tissue insulin sensitivity of iWAT and eWAT was assessed at the end point via injections of a bolus of insulin (INS) (+), which was followed by rapid tissue dissections and extraction 15 min later. Signaling induction was assessed via Western blotting for pAKT (Ser473 and Thr308). Control groups were injected with saline (−). WBs were quantified by Image J image analysis. pAKT (Ser473 and Thr308) were both engaged in iWAT of CD‐fed mice upon insulin injections. This difference was significant and disappeared on HFD. Significantly increased pAKT/(Ser473)AKT was also detected in eWAT of CD and HFD‐fed mice, but no Thr308 phosphorylation was observed. Error bars represent SD. *n* = 3. Statistical significance is represented as *p*‐values.

**FIGURE 5:**
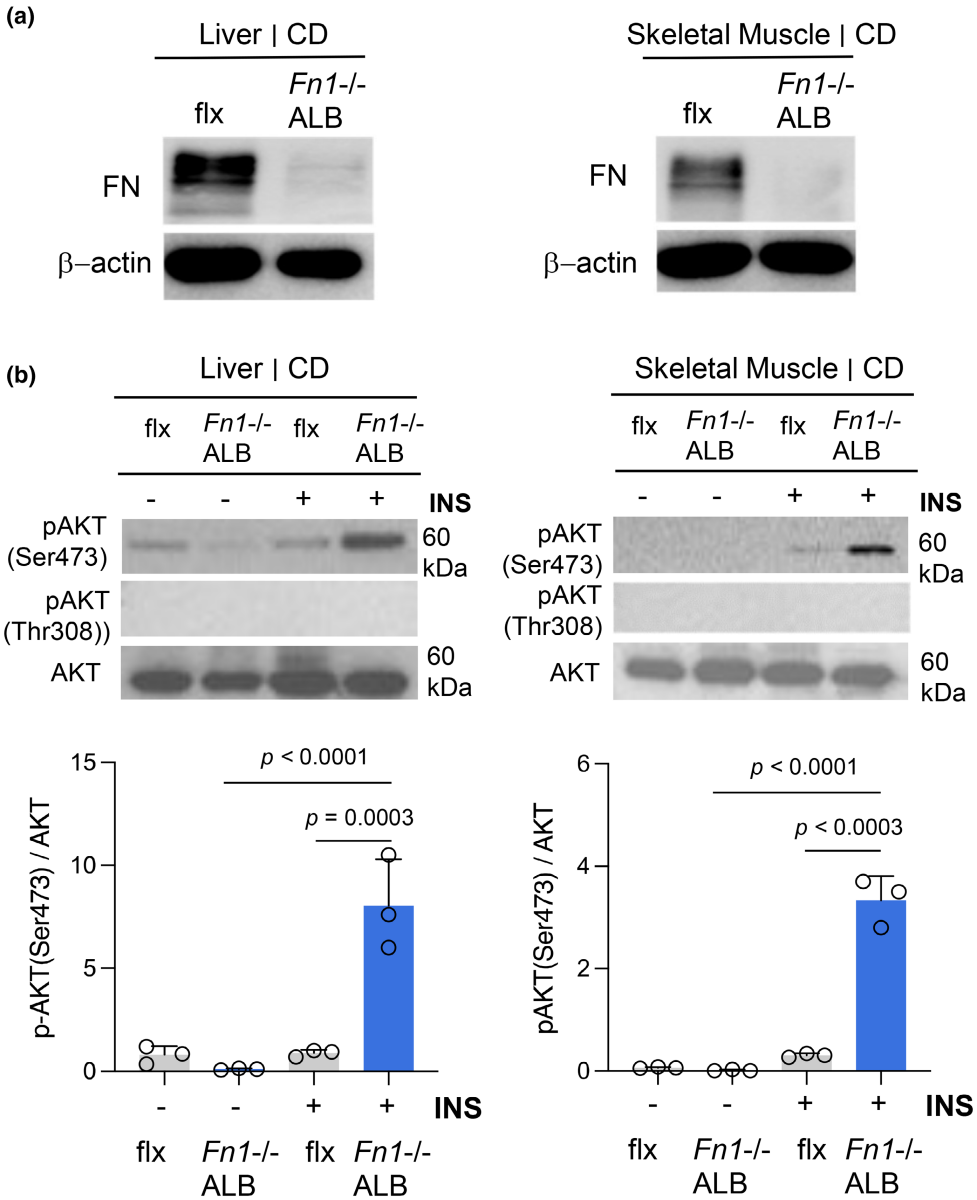
The absence of pFN significantly increases in vivo insulin sensitivity of liver and skeletal muscle of CD diet fed male mice. (a) WB analysis of total FN levels in the liver and skeletal muscle of flx/flx and *Fn1*−/−ALB mouse. As expected, the liver of the knockout mouse shows no FN. Skeletal muscle shows no presence of FN suggesting that the pFN contributes to majority of its FN matrix. β‐Actin was used as loading control. (b) In vivo tissue insulin sensitivity of liver and skeletal muscle was assessed at the end point via injections of a bolus of insulin (INS) (+), which was followed by rapid tissue dissections and extraction 15 min later. Signaling induction was assessed via Western blotting for pAKT (Ser473). Control groups were injected with saline (−). WBs were quantified by NIH Image J image analysis. pAKT (Ser473) was significantly engaged in both tissues upon insulin injections. Error bars represent SD. *n* = 3. Statistical significance is represented as *p*‐values.

Analysis of fasting and resting glucose at the endpoint of the 20‐week feeding showed no significant differences in male knockout mice compared to their controls (Figure S4). However, the glucose tolerance test (GTT) showed significantly better glucose clearance in male *Fn1*−/−ALB mice on *normal CD‐feeding* regime in comparison to the controls (Figure 4a). The difference was not detected HFD (Figure 4a). Female mice did not show any differences in fasting glucose or in GTT assay (Figure S5A). Insulin tolerance test (ITT); to probe whole‐body insulin sensitivity showed significant enhancement in male *Fn1*−/−ALB mice compared to control mice *on normal CD‐feeding*, which was supported by kITT value (plasma glucose disappearance rate in 30 min), which showed significantly higher glucose elimination rate in *Fn1*−/−ALB mice (Figure 4b). The kITT value reflects tissue insulin sensitivity well, as at the 30‐min time point hyperglycemia‐induced counter regulatory hormones have not exerted their action (Monzillo & Hamdy, 2003; Rizza et al., 1979). The increased insulin sensitivity difference was not detected on HFD (ITT and kITT) using same amount of insulin as on CD. Insulin sensitivity in HFD‐fed mice is often tested with higher insulin amounts due to developing insulin resistance; however, the higher levels of insulin induced hypoglycemia and it was not possible to complete the test for proper conclusions. However, since the glucose clearance did not show differences on HFD, it is likely that the increased insulin sensitivity on CD is lost on HFD. No changes were observed in glucose clearance or insulin sensitivity in female mice (Figure S5B,C). In vivo insulin sensitivity assay of iWAT and eWAT with bolus of insulin injections, using higher insulin amount, followed by rapid dissections, tissue extraction and signaling analysis by WB, supported the observed differences on normal diet. The iWAT of male *Fn1*−/−ALB mice in the CD‐fed group showed significantly increased phosphorylation of AKT to both Ser473 and Thr308 residues (Figure 4c). The difference was not observed in the HFD‐fed group (Figure 4c). eWAT insulin sensitivity, pAKT(Ser473)/AKT was similarly significantly increased in the knockout mice and the effect remained significant also on HFD (Figure 4d), however, no phosphorylation to Thr308 was detected in eWAT. Considering the accrual of pFN to other metabolic tissues, we also tested the levels of FN and in vivo insulin sensitivity of liver and skeletal muscle of the *Fn1*−/−ALB mice. As expected, WB analysis of FN in liver extract showed no FN detection in the knockout, and interestingly also skeletal muscle showed no presence of FN in the knockout tissue (Figure 5a). Interestingly, both showed a significant increase in AKT phosphorylation (Figure 5b) under the same experimental setup as WAT, suggesting FN may regulate insulin sensitivity via a similar molecular mechanism.

**FIGURE 6:**
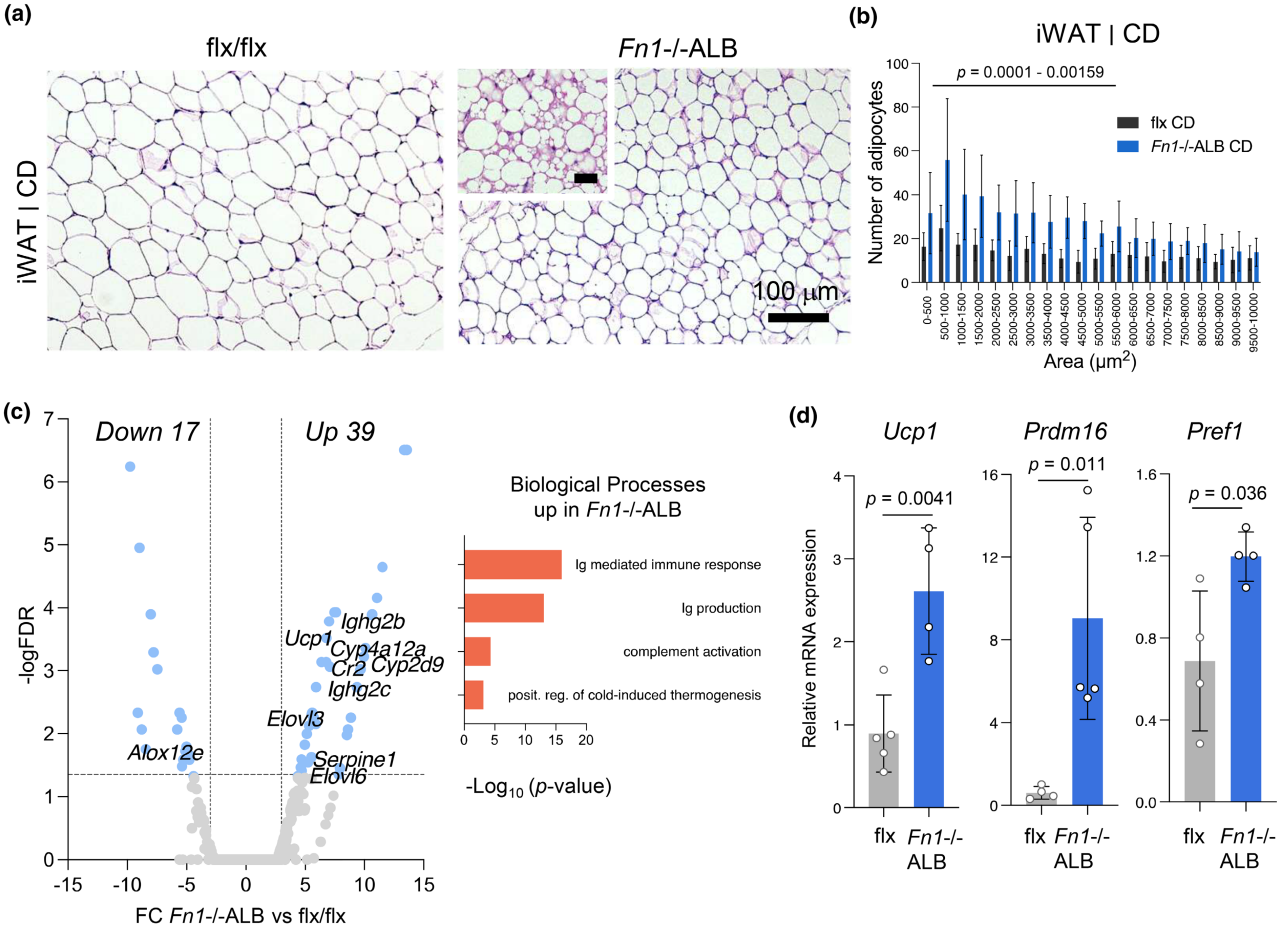
iWAT of control diet‐fed *Fn1*−/−ALB mice shows increased cellularity and presence of beige adipocytes. (a, b) H&E‐stained histological sections and quantification of adipocyte cell sizes in iWAT of control diet (CD)‐fed mice show smaller adipocytes in *Fn1*−/−ALB mice with pockets of high cellularity (insert). Small adipocytes with area <6000 μm^2^ (<~80 μm diameter) were significantly increased in the *Fn1*−/−ALB. (c) Differential gene expression and gene enrichment analysis from RNA sequencing analysis of iWAT of CD‐fed *Fn1*−/−ALB and flx mice. Annotated genes in the volcano plot represent gene ontology terms. Analysis revealed upregulated pathways including cold‐induced thermogenesis suggesting the presence of beige adipocytes. (d) qPCR analysis of preadipocyte (*Pref1*) and beige adipocyte markers (*Ucp1*, *Prdm16*) showed a significant increase in *Fn1*−/−ALB iWAT. Error bars represent SD (*n* = 4–5 mice per group). Statistical significance is represented as *p*‐values between knockout and its control.

To get insight also at tissue level into how the absence of pFN affects the AT depots on both diets, histological analysis of iWAT and eWAT from CD and HFD‐fed male mice was done jointly with RNA sequencing analysis. Analysis of iWAT from CD‐fed *Fn1*−/−ALB mice revealed visibly smaller cells in the knockout tissue, which was confirmed to be significant by cell size quantification (Figure 6a,b). Pockets of high cellularity were also observed (Figure 6a, inset). iWAT from CD‐fed mice had a total of 56 differentially regulated genes (DEGs) which majority were upregulated (Figure 6c). Gene enrichment analysis (GEA) of the upregulated genes using Metascape and confirmation with Panther and KEGG revealed biological processes related to immunity and positive regulation of cold‐induced thermogenesis suggesting browning/beiging of iWAT (Figure 6c) (GEA of downregulated genes did not result in any consensus Gene Ontology (GO) terms). Genes contributing to this GO term included *Elovl3*, *Ucp1*, *Elovl6*. *Ucp1* (Uncoupling Protein 1) is a key thermogenesis protein and showed 7‐fold upregulation in the RNAseq data. These data prompted us to examine adipocyte differentiation markers by qPCR (Figure 6d) which showed significant increase in preadipocyte marker, *Pref1*, *Ucp1*, and beige adipocyte precursor marker *Pdrm16* (Figure 6d) suggesting increase in beige adipocytes in the iWAT of CD‐fed *Fn1*−/−ALB mice.

**FIGURE 7:**
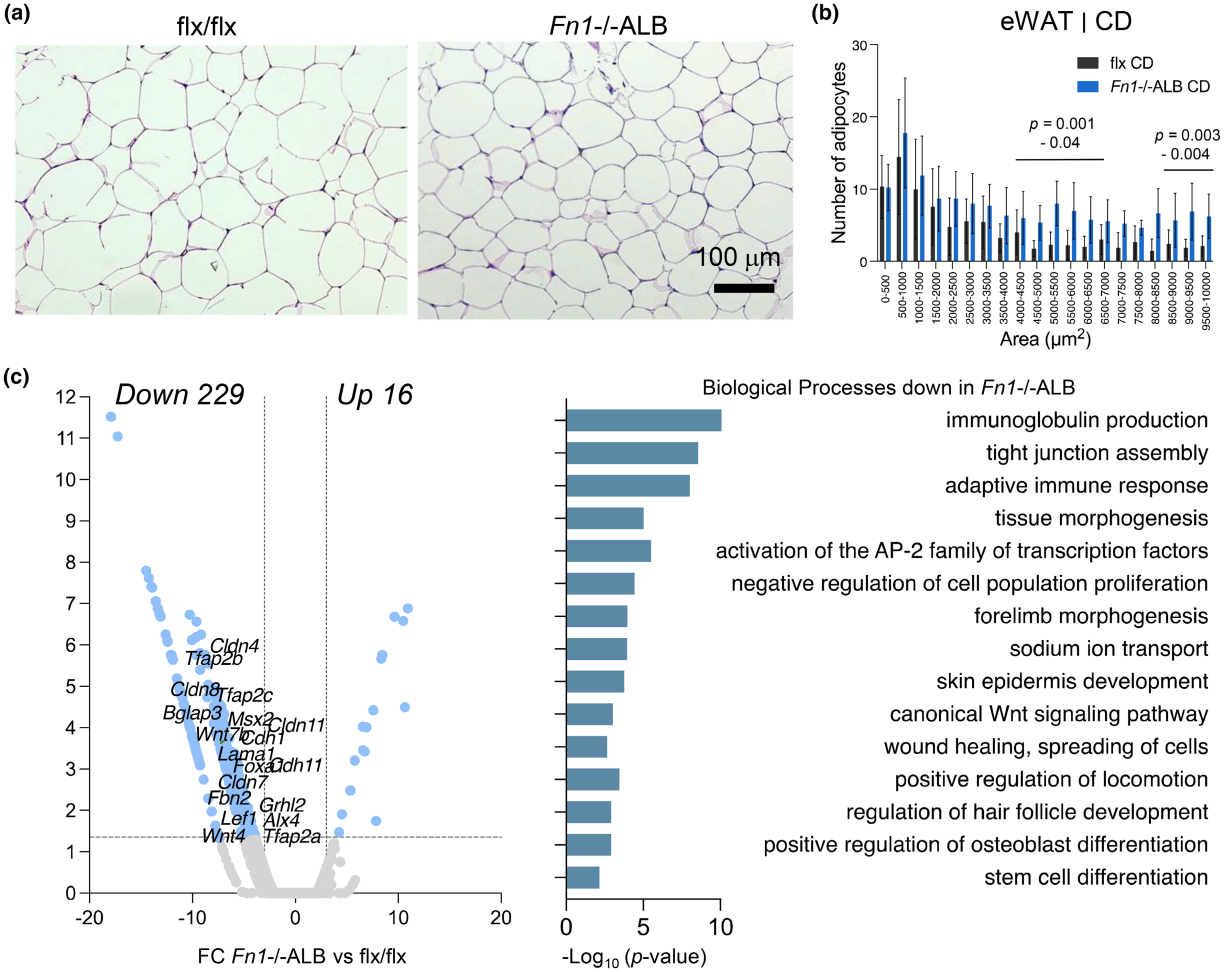
eWAT of control diet‐fed *Fn1*−/−ALB mice show decreased expression of genes related to development and stem cell differentiation. (a, b) H&E‐stained histological sections and quantification of adipocyte cell sizes in eWAT of CD‐fed mice show moderate alterations of cell sizes with a significant increase in the larger in *Fn1*−/−ALB mice (area >8000 μm^2^ (<~100 μm diameter). (c) Differential gene expression and gene enrichment analysis from RNA sequencing data of eWAT of CD‐fed *Fn1*−/−ALB and flx mice. Annotated genes in volcano plot represent gene ontology terms. Analysis revealed downregulation of genes related to tissue morphogenesis, development, and stem cell differentiation. (d) Error bars represent SD (*n* = 4–5 mice per group). Statistical significance is represented as *p*‐values between knockout and its control.

Adipocyte sizing and RNA sequencing analysis from eWAT of the CD‐fed *Fn1*−/−ALB mice demonstrated also major changes. The tissue had a significant increase in the larger adipocytes compared to control group (Figure 7a). RNA sequencing identified 245 DEGs of which 229 were downregulated (Figure 7c). DEA identified several downregulated biological processes of which notable were activation of AP‐2 family of transcription factors (*Tfab2a*, *Tfab2b*, *Tfab2c*), canonical Wnt signaling (e.g., *Wnt7b*, *Wnt4*, *Lef1*, *Dkkl1*), as well as tissue and forelimb morphogenesis representing collectively development and suggesting changes in mesenchymal stem cell populations and/or their differentiation.

**FIGURE 8:**
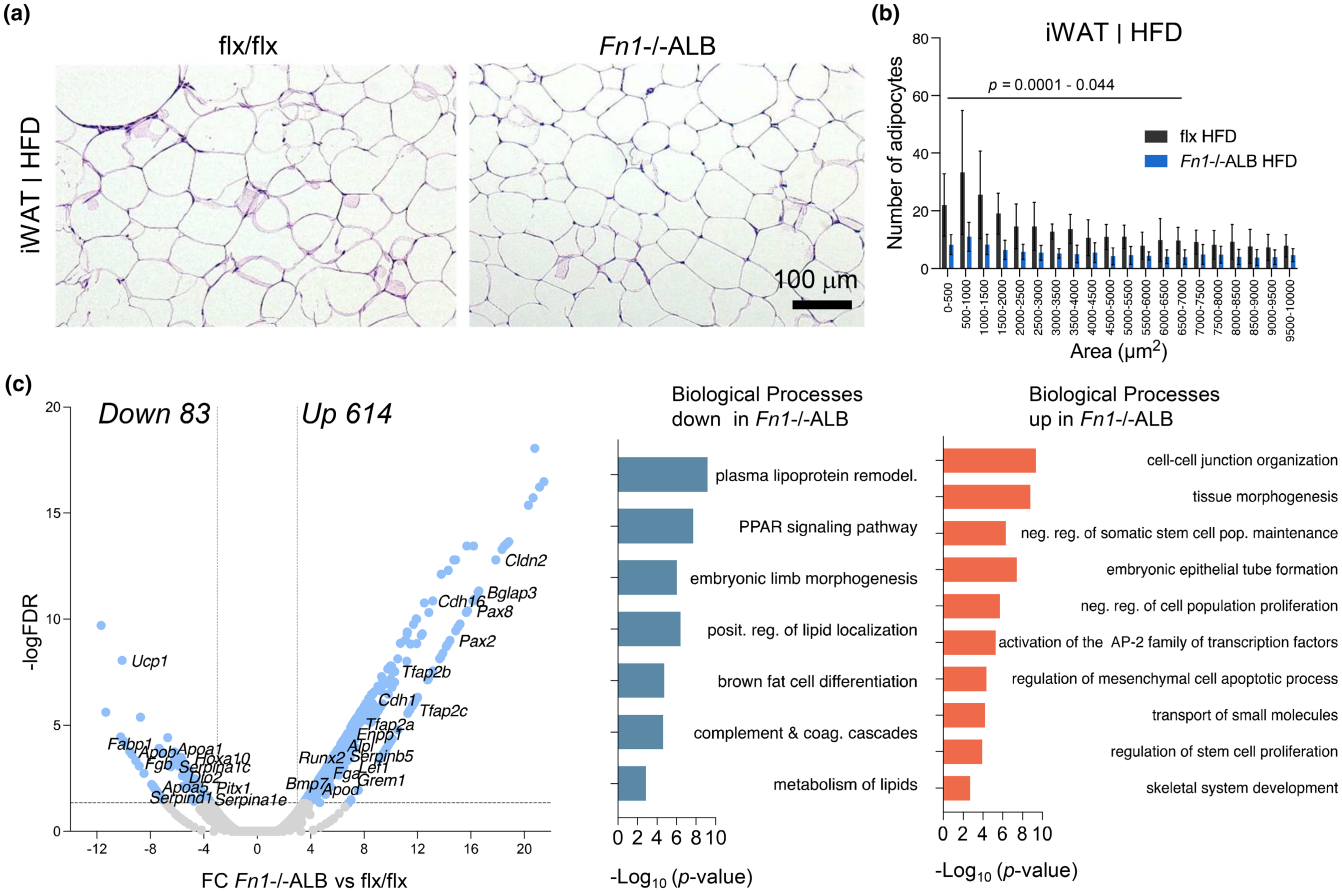
iWAT of HFD‐fed *Fn1*−/−ALB mice show alterations in lipid metabolism, adipogenesis and stem cell maintenance. (a, b) H&E‐stained histological sections and quantification of adipocyte cell sizes in iWAT of HFD‐fed mice show normal expansion of adipocytes *Fn1*−/−ALB but a significant decrease of smaller adipocytes under area <7000 μm^2^ (< ~95 μm diameter). (c) Differential gene expression and gene enrichment analysis from RNA sequencing analysis of eWAT of HFD‐fed *Fn1*−/−ALB and flx mice. Annotated genes in volcano plot represent gene ontology terms. Analysis revealed upregulation of genes related to tissue morphogenesis, development and mesenchymal stem cell differentiation. Downregulated genes represented adipocyte lipoprotein remodeling, lipid metabolism and adipocyte differentiation. Error bars represent SD (*n* = 4–5 mice per group). Statistical significance is represented as *p*‐values between knockout and its control.

Analysis of the iWAT depot of the HFD‐fed *Fn1*−/−ALB mice also alterations in adipocyte sizes and transcription. Histological analysis and quantification of the adipocyte sizes showed comparable adipocyte expansion as in control tissue but a significant reduction in the smaller cell pools (Figure 8a,b). RNA sequencing data (Figure 8c) showed 697 DEGs in *Fn1*−/−ALB of which 614 were upregulated, and DEA resulted in biological processes linked to tissue morphogenesis, activation of AP‐2 family of transcription factors (*Tfab2a*, *Tfab2b2*, *Tfab2c*), stem cell proliferation, and skeletal system development represented by transcription factors *Runx2*, *Grem1* and mineralization regulators *Alpl*, *Enpp1*, *Bglap3*. Downregulated biological processes were linked to plasma lipoprotein remodeling (e.g., *Fabp1*, *Apoa1*, *Apoa5*, *Apob*), PPAR signaling, and brown fat cell differentiation (*Ucp1*, *Dio2*).

**FIGURE 9:**
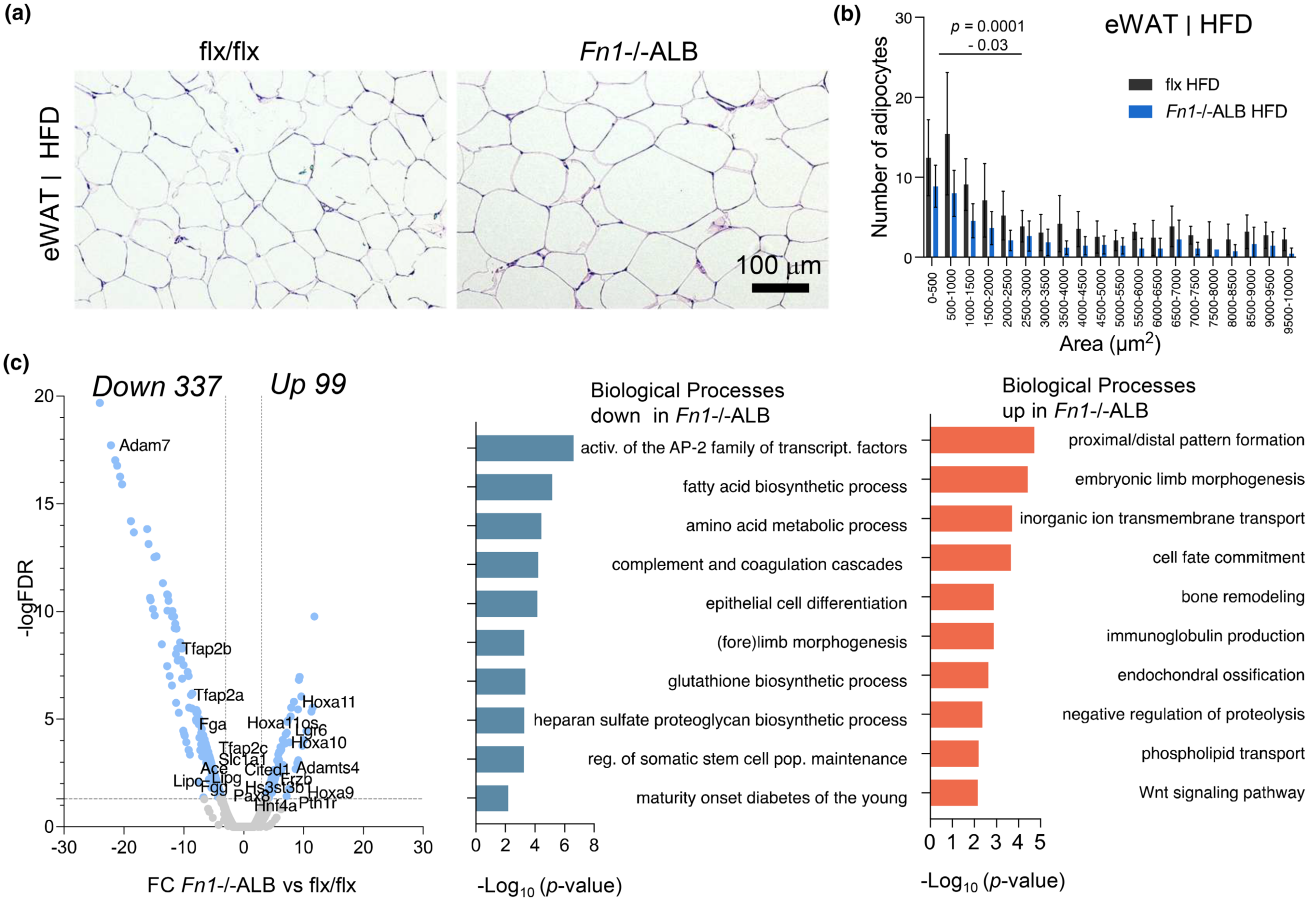
eWAT of HFD‐fed *Fn1*−/−ALB mice show a decrease in small adipocytes and alterations stem cell differentiation. (a, b) H&E‐stained histological sections and quantification of adipocyte cell sizes in eWAT of HFD‐fed mice show normal expansion of adipocyte size in *Fn1*−/−ALB mice with a significant decrease in the pool of small adipocytes with area of <3000 μm^2^ (<~60 μm diameter). (c) Differential gene expression and gene enrichment analysis from RNA sequencing analysis of eWAT of HFD‐fed *Fn1*−/−ALB and flx mice. Annotated genes in volcano plot represent gene ontology terms. Analysis showed downregulation in genes representing adipocyte lipoprotein remodeling, lipid metabolism and adipocyte differentiation and upregulation of transcription factors related to adult stem cell differentiation, notably those related to skeletal morphogenesis. Error bars represent SD (*n* = 4–5 mice per group). Statistical significance is represented as *p*‐values between knockout and its control.

The eWAT of the HFD‐fed *Fn1*−/−ALB analysis showed that adipocytes had expanded similarly as control adipocytes, however a decrease in small adipocyte pools was detected compared to control group (Figure 9a,b). RNA sequencing data (Figure 9c) showed 436 DEGs of which 337 were downregulated. DEA linked the downregulated genes to biological processes of activation of AP‐2 family of transcription factors (*Tfab2a*, *Tfab2b2*, *Tfab2c*), fatty acid biosynthesis (e.g., *Lipc*, *Lipg*), regulation of somatic (adult) stem cell population maintenance (e.g., *Pax8*, *Hnf1b*, *Trp63*, *Hnf4a*, *Pklr*) and maturity onset of diabetes of the young (*Hnf4a*, *Pklr*, *Hnf1b*). In this analysis the upregulated gene set resulted in links to biological processes of embryonic limb morphogenesis, bone remodeling and endochondral ossification (e.g., *Pth1r*, *Ptn*, *Wnt16*, *Frzb*, *Adamts4*) as well as Wnt‐signaling (e.g., *Lgr5*, *Frzb*, *Wnt16*, *Lgr6*) were highly represented. Collectively these suggest alteration in mesenchymal stem cell pools and/or regulation.

## 4 DISCUSSION

Extracellular matrix is an important regulator of adipogenesis and AT cellular homeostasis with effects on whole‐body metabolism (DeBari & Abbott, 2020; Kaartinen et al., 2022; Lin & Kang, 2016; Ruiz‐Ojeda et al., 2019; Williams et al., 2015). Generally, ECM components are synthesized locally in AT by the adipocyte lineage cells as well as other AT cell types. Here we provide evidence that a circulating ECM protein, pFN, accumulates to AT and contributes to the homeostasis, insulin sensitivity, and cell pools of the tissue. The role of pFN as a major source of tissue ECM in vitro and in vivo was first introduced over 40 years ago by Oh et al. (1981) and further elaborated in several studies including Sottile & Hocking (Sottile & Hocking, 2002) who demonstrated that *Fn1*−/−MEF cultures can assemble normal FN matrix from the serum pool of FN. A study by Moretti et al. (2007) further demonstrated that mice with hepatocytes genetically engineered to produce EDA domain in *Fn1* gene exhibited a significant reduction in FN levels in both plasma and tissues. Conversely, when liver‐specific deletion of the EDA‐domain was removed again in these same animals, FN levels in plasma and tissues (skin, kidney, liver, heart muscle, lung, and brain) were restored. This study emphasized the pFN contribution to tissue ECM and showed that FN harboring EDA‐domain cannot be secreted from hepatocytes (Moretti et al., 2007). The development of *Fn1*flx/flx mouse (Sakai et al., 2001) has allowed for further understanding of pFN in different systems via hepatocyte‐specific *Fn1* knockouts (with ALB‐Cre and inducible MX1‐Cre), and its role has been linked to supporting cell survival and tissue integrity such as in brain injury (Sakai et al., 2001), normal bone matrix integrity (Bentmann et al., 2010), and vascular development (Kumra et al., 2018) but no roles were identified in skin healing or hemostasis. Our data provides AT to this list as a tissue where pFN accumulates and has a function.

In vitro evidence has long suggested that the total FN matrix (source was not defined, can be cFN or/and pFN) sustains preadipocyte phenotype and inhibits adipogenesis via binding to Pref1 (Wang et al., 2010). It was demonstrated that, FN fragments containing I_1‐5_, such as 24 k FibI, promote adipogenesis by blocking FN fibrillogenesis and matrix assembly (Chernousov et al., 1991; Fukai et al., 1993; Kamiya et al., 2002). Our previous work demonstrated that pFN from cell culture serum, jointly with transglutaminase FXIII‐A from preadipocytes, can maintain pre‐adipocyte phenotype, that is, the transglutaminase stabilization step promoted the creation of the pFN matrix. Inhibiting FXIII‐A activity reduced pFN matrix and modulated cytoskeletal dynamics and resulted in different responses from preadipocytes to insulin in a manner where in the presence of pFN cells responded with proliferation and the absence of pFN jointly with insulin promoted differentiation (Myneni et al., 2014). These data have collectively suggested an important role for FN and pFN in AT. In the current study, we provide in vivo evidence that pFN accumulates to normal WAT, its levels being particularly high in eWAT. In iWAT pFN is clearly the main FN isoform as pFN KO mice show very low detection of FN in iWAT, that is., a decrease of −86% of the FN matrix of control tissue. The observation is similar to reports on other tissues where pFN was shown to contribute to nearly all detectible FN matrix, such as bone (Bentmann et al., 2010). pFN was also found accumulated in other metabolic tissues including skeletal muscle. Interestingly, no FN was detected in the skeletal muscle of the pFN knockout (compared to control that had abundant FN detection) as per to additional WB analysis showing the important contribution of plasma FN pool to its FN matrix. In our pFN injection work, we used only a fluorescence control as identifying an appropriate hepatocyte‐derived and/or circulating large protein control was difficult. Fibrinogen was considered as most suited as it has functional relation to fibronectin, however, its accumulation to AT has also been reported at least on HFD, where it functions to promote AT inflammation (Kopec et al., 2014, 2017).

Our data from the pFN knockout shows that pFN appears to have a metabolic role in *normal* WAT, that is, on normal (control) diet‐fed male mice. pFN absence had no significant effect on weight gain or on fat%, fat mass, adiposity or AT depot volumes on HFD‐feeding regime. However, the absence of pFN caused a significant enhancement on whole‐body insulin sensitivity and promoted AT, liver and skeletal muscle insulin sensitivity on *normal diet*. iWAT showed a presence of small adipocytes, and most interestingly, beige adipocytes which are highly insulin sensitive (Bartelt & Heeren, 2014; Lizcano, 2019; Tandon et al., 2018). eWAT of *Fn1*−/−ALB mice remained more insulin sensitive after HFD feeding suggesting that pFN may contribute to the AT depot homeostasis differently. The differences were also seen in the result that only in iWAT pAKT phosphorylation occurred to Thr308, which may lead to activation of different pathways. The observation that absence of pFN enhanced also liver and skeletal muscle insulin sensitivity suggests a common molecular mechanisms. Our previous work demonstrated that pFN can potentiate the pro‐proliferative effects of insulin via insulin receptor (IR) but not via insulin‐like growth factor receptor (IGFR) (Myneni et al., 2014). The effects of pFN on insulin signaling may be direct or mediated by integrin engagement which is known to boost insulin sensitivity in a manner where β1/β3 integrin activation contributes to insulin sensitivity and browning/beiging of AT (Ruiz‐Ojeda et al., 2021). pFN may also affect other components of ECM and influence the differentiation of other AT cells in a manner that more insulin‐sensitive cells are prominent. Interestingly, exercise‐induced myokine, irisin (fibronectin‐type III domain‐containing protein 5) interacts with αVβ1/β5‐integrins to induce WAT browning and beige adipocyte biogenesis (Aladag et al., 2023; Oguri et al., 2020). Whether there is a link between irisin and pFN is not known.

Our study also demonstrated that the absence of pFN influences the transcriptional landscape of the AT which reflected development, morphogenesis and genes of mesenchymal stem cells. The absence of pFN affected eWAT on normal diet via causing downregulation of genes related to skeletal development and stem cells most notably Wnt‐signaling, and AP‐2 transcription factor family regulation (*Tfab2a*, *Tfab2b2*, *Tfab2c*). HFD induced even more substantial changes to the transcriptional landscape with hundreds of upregulated genes in iWAT, which also related to tissue morphogenesis, mesenchymal stem cell population maintenance, skeletal system (*Runx2*, *Grem1*, *Alpl*, *Bglap3*) and AT‐2 transcription factors. The downregulated pool in turn reflected adiposity including plasma lipoprotein remodeling with several apolipoprotein downregulation (*Apoa1*, *Apoa2*, *Apob*, *Apoa5*) and brown fat cell differentiation (*Ucp1*, *Dio2*). Interesting changes were also seen in HFD‐fed eWAT of *Fn1*−/−ALB eWAT gene expression with downregulation of skeletal development‐related transcription factors, including *Grem1*; *Runx2*, *Lef1*; mineralization regulators, *Enpp1*, *Pitx1*, *Bglap3*, and osteogenesis related, *Pthr1* (parathyroid hormone 1 receptor) suggesting that adipose stem cells may have altered phenotypes in the absence of pFN. Also, tube morphogenesis was altered where fibronectins have been shown to have a major role (Astrof & Hynes, 2009; Hendel & Granville, 2013). Insulin receptor signaling has been demonstrated to regulate pluripotency and differentiation of stem cells (Belfiore et al., 2017; Godoy‐Parejo et al., 2019; Gupta et al., 2018; White & Kahn, 2021) via Wnt/beta‐catenin pathways (Freund et al., 2008; Lian et al., 2013). Interestingly, AP‐2α and AP‐2β transcription factors mediate craniofacial development via Wnt pathway (Van Otterloo et al., 2022), and AP‐2β can regulate adipocyte enlargement and insulin sensitivity in vitro (Tao et al., 2006). Considering the essential role of *Fn1* gene in early development and its importance to stem cell adhesion (Lukjanenko et al., 2016), it is not all surprising to see the links to the stem cell and developmental genes; however, links of fibronectins to insulin signaling in this context have not been explored.

While the circulating pool of pFN serves as a source for the tissue ECM, our data suggested that the actual assembly is promoted locally by the presence and activity of FXIII‐A transglutaminase (Myneni et al., 2014, 2016). pFN is a well established substrate of FXIII‐A (Keski‐Oja et al., 1976; Mosher, 1975, 1977, 1978; Mosher et al., 1980; Mosher & Schad, 1979), which is best known for its function as fibrin‐stabilizing factor at the last step of the coagulation cascade (Komaromi et al., 2011; Mitchell & Mutch, 2019; Muszbek et al., 2011). The circulating plasma FXIII‐A is produced in the bone marrow by monocytes, macrophages and megakaryocytes (Griffin et al., 2015; Malara et al., 2011); however, recent advances from us and others have established its production also in tissues by several cell types (Mitchell & Mutch, 2019). It is conceivable to consider that both the availability of pFN in plasma and tissue FXIII‐A levels and transglutaminase activity jointly affect the pFN ECM in the tissues (Malara et al., 2011; Myneni et al., 2014, 2016). We have also reported this FXIII‐A‐mediated assembly mechanism in mesenchymal stem cells and osteoblast cultures where pFN and FXIII‐A promote osteoblastogenesis in vitro. In vivo, elimination of FXIII‐A together with another TG, TG2, affects bone and marrow pFN levels, bone remodeling and mass in vivo (Cui et al., 2014; Mousa et al., 2017). Similarly, Malara et al. (2011) demonstrated that megakaryocyte FXIII‐A promotes pFN assembly in the bone marrow. Interestingly, in this current study, pFN absence in iWAT was associated with decreased FXIII‐A levels in iWAT, but not in plasma, likely because of different cellular source of the enzyme. In AT, FXIII‐A is likely produced by preadipocytes, but also likely by hypertrophic adipocytes as suggested by our recent studies in humans (Kaartinen et al., 2005, 2020, 2022). Thus, it is possible that pFN/FXIII‐A ECM at different stages of adipogenesis may result in different effects, for example on preadipocytes/precursors in iWAT and hypertrophic adipocytes in eWAT.

The functions of liver and AT are tightly connected in obesity. Functional failure of AT results in exogenous triglyceride accumulation in liver (nonalcoholic fatty liver disease, NAFLD) (Stefan et al., 2023). Hepatokines in turn, such as fibroblast growth factor‐21 and fetuin‐A, regulate AT function and insulin sensitivity. NAFLD, cirrhosis and alcoholic liver also affect pFN levels (Matsuda et al., 1982; Mosher, 1986). Our study suggests that pFN may also act as a hepatokine, and liver‐metabolism mediator. In humans, data on circulating pFN levels in obese individuals, with and without NAFLD has been reported with no clear consensus (Cucuianu et al., 1996; Dejgaard et al., 1984; Gluud et al., 1983; Matsuda et al., 1982). pFN levels were shown higher in overweight males versus females with the same body mass index (BMI) and the levels increased with age (Cucuianu et al., 1996; Delpuech et al., 1988; Ekaidem et al., 2011). Andersen et al. (1987) demonstrated that obesity increases FN levels in circulation and weight loss returns the levels to normal. Other reports documented that type 2 diabetic patients showed lower circulating pFN levels than controls of the same age groups and that the levels showed no difference between females and males (Labat‐Robert et al., 1984). In these studies, it was not defined if circulating FN was the ‘classic pFN’ or cellular, EDA‐FN (from tissues) which was recently linked to systemic inflammation and insulin resistance via activation of Toll‐like receptor 4 (TLR4) (Malara et al., 2019; Rajak et al., 2020).

In summary, our data expands and deepens our understanding of the role of FN matrices in AT, metabolism and whole‐body health and suggests a function for pFN in regulation of tissue insulin sensitivity and adipocyte differentiation and stem cell maintenance of adipose tissue in vivo. Modulation of its levels may benefit overall metabolic health.

## AUTHOR CONTRIBUTIONS

M.M., M.T.K. designed study; M.M., E.M.D., M‐H.N., Y.P., A.G., M.M., S.C., conducted the experimental work; M.M., E.M.D., M.T.K., L.M. analyzed and interpreted data; M.M., M.T.K., drafted manuscript; and M.T.K, L.M., edited and revised manuscript. All authors approved the final content of the manuscript.

## FUNDING INFORMATION

This work was supported by grants from the Canadian Institutes of Health (PJT‐15089, PJT‐162100) to MTK and by grants from the Canadian Institutes of Health (PJT‐175306, PJT‐162302), and the Alzheimer's Association US AARG‐21‐852,152 to LM. MTK is a member of Le Fonds de recherche du Quebec—Santé (FRQS) Network for Oral and Bone Health (RSBO) and FRQS Cardiometabolic Health, Diabetes and Obesity Research Network (CMDO). MM is supported by stipend from the Faculty of Dental Medicine and Oral Health Sciences and RSBO. EDM is supported by a stipend from FRQS. YP received the Davis fellowship through McGill's Faculty of Medicine and a stipend from the Canada First Research Excellence Fund, awarded to McGill University for the Healthy Brains for Healthy Lives initiative and is supported by a stipend from FRQS.

## CONFLICT OF INTEREST STATEMENT

The authors declare no conflicts of interest and there are no financial conflicts to disclose.

## ETHICS STATEMENT

The animal care and experimental procedures were under the Canadian Council of Animal Care and approved by the McGill University Animal Care Committee (protocol code MCGL‐5188, May 3, 2023).

## Supporting information


Figures S1–S5.

## Data Availability

Data is available upon request.
