# Supplementary material for: Circulating plasma fibronectin affects tissue insulin sensitivity, adipocyte differentiation, and transcriptional landscape of adipose tissue in mice
Source: Physiol Rep. 2024 Jul 25;12(14):e16152. doi: 10.14814/phy2.16152 (PMC11272447; doi:10.14814/phy2.16152)
Supplement: Supplementary file 1 — Figures S1–S5. [file PHY2-12-e16152-s001.pdf]

Supplemental Figure 1

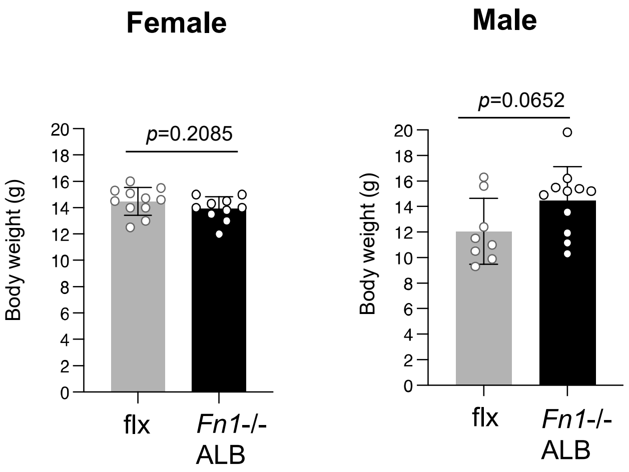

Supplemental Figure 1. Body weight of 4-week-old male and female mice prior to starting the CD or HFD feeding regime. Body weights were not significantly different at 4-week-age between knockout and its control. Error bars represent SD (n=9-11 per group). Statistical significance was defined as $p<0.05$.

Supplemental Figure 2

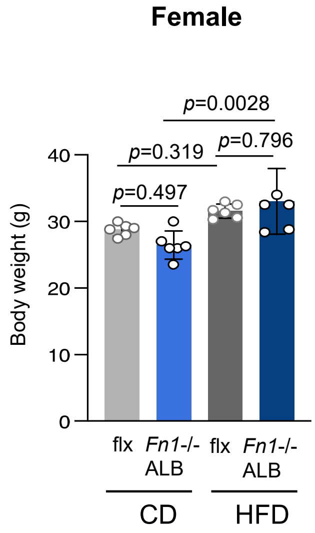

Supplemental Figure 2. Body weights of female *Fn1*-/-ALB and its control flx mice after 20 weeks on the control diet (CD) and high-fat diet (HFD). No significant difference was observed between the knockout and its control. HFD did not induce significant weight gain in flx mice, but significant weight gain was observed in *Fn1*-/-ALB. Error bars represent SD (n=5-6 per group). Statistical significance was defined as $p<0.05$.

Supplemental Figure 3

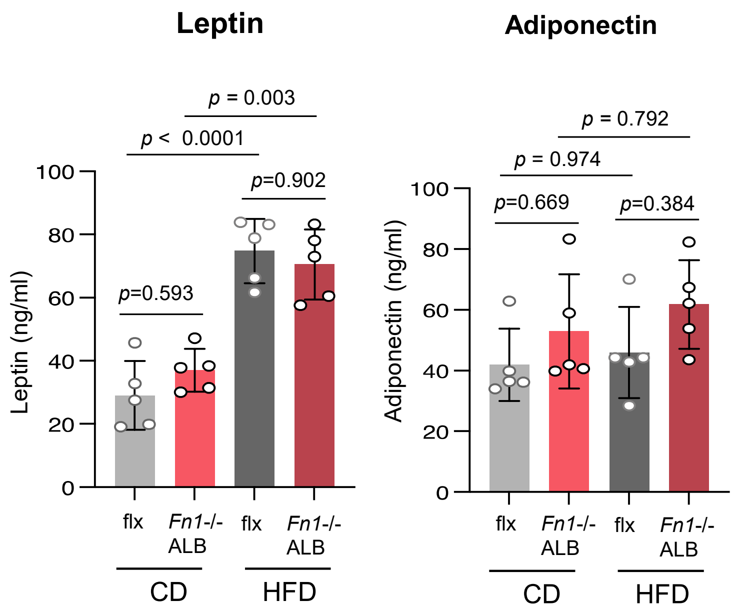

Supplemental Figure 3. Serum leptin and adiponectin levels in male *Fn1*-/-ALB and flx mice. Both markers do not show significant change between the *Fn1*-/-ALB and flx control mice on control diet (CD) or high-fat diet (HFD). HFD increased serum leptin levels significantly in both mouse models on HFD reflecting the increased adiposity. Error bars represent SD (n=5 per group). Statistical significance was defined as $p<0.05$.

Supplemental Figure 4

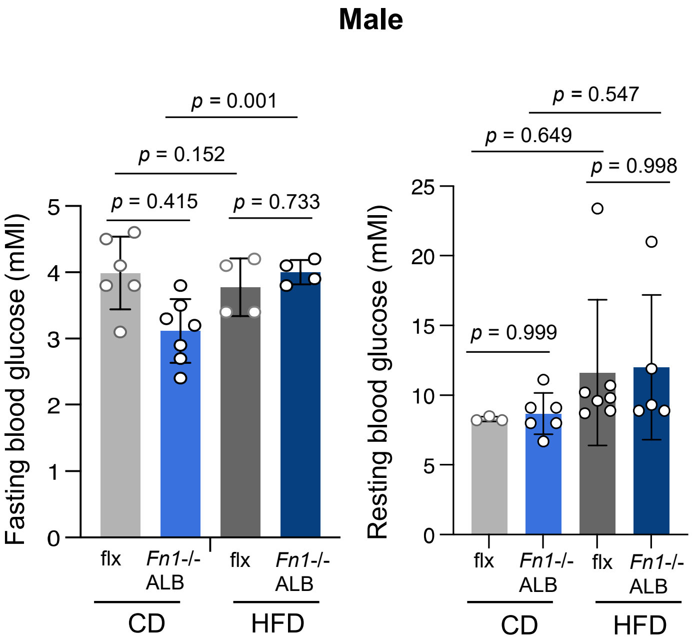

Supplemental Figure 4. Fasting and resting glucose levels of male and female *Fn1-/-*-ALB and control mice. Fasting (6 h) glucose levels did not show significant differences in male mice on control diet (CD) or high-fat diet (HFD) albeit the HFD-induced increase in fasting blood glucose was clearer and significant in the knockout mice. Error bars represent SD (n=4-7 per group). Statistical significance was defined as $p<0.05$.

Supplemental Figure 5

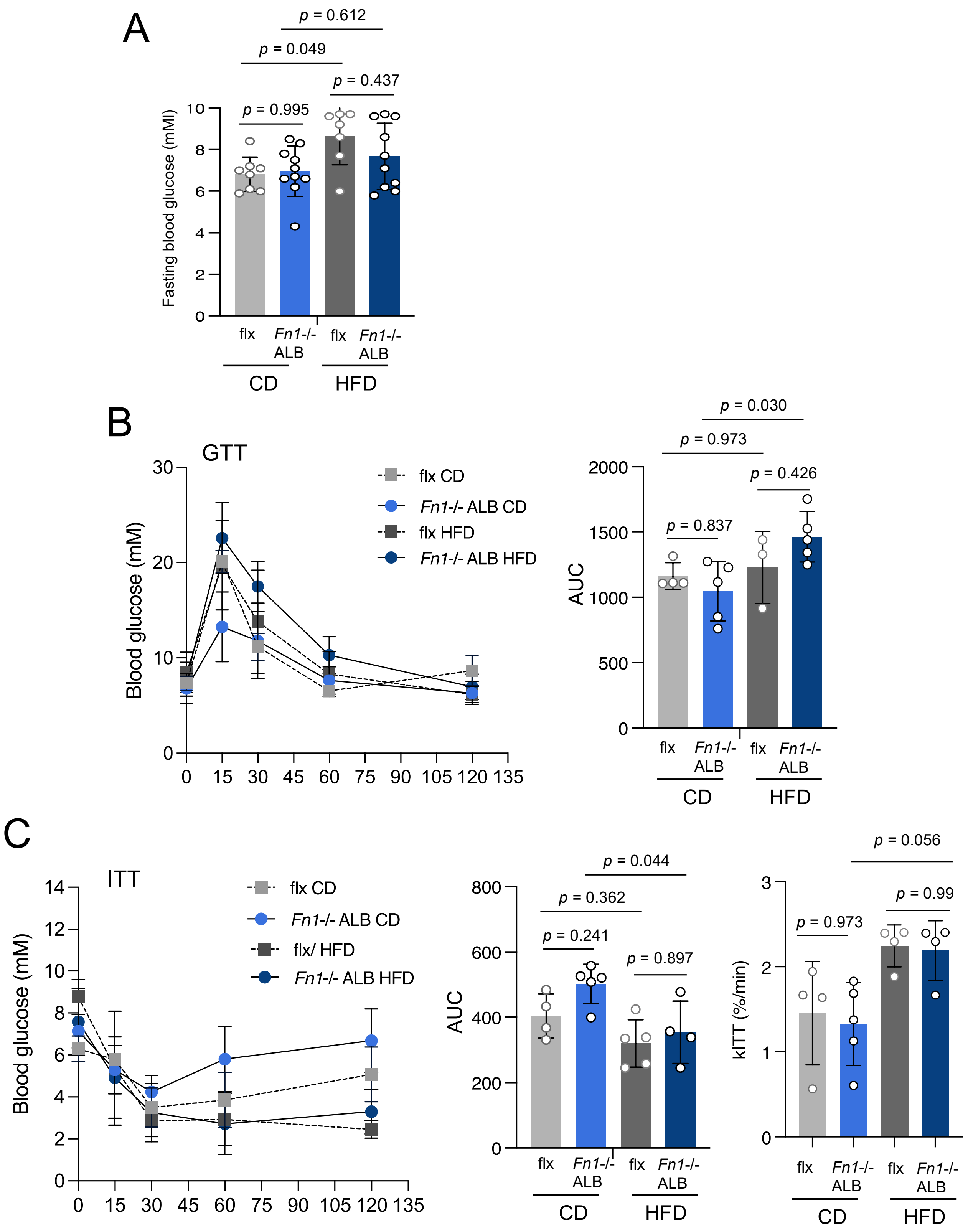

Supplemental Figure 5. Fasting glucose levels, whole-body glucose clearance and Insulin sensitivity in female *Fn1-/-*-ALB mice. A. Fasting glucose levels show no significant difference between the knockout and controls on control diet (CD or high-fat diet (HFD), **B.** Glucose clearance (glucose tolerance test, GTT) and **C.** Insulin sensitivity (insulin tolerance test; ITT and kITT; decrease of glucose [%)] per minute) on CD or HFD show no significant differences between the knockout and control mice. Error bars represent SD (n=3-8 per group). Statistical significance was defined as $p<0.05$.
